# Host life history traits as drivers of viral infection in European bats

**DOI:** 10.1016/j.virusres.2026.199821

**Published:** 2026-10-23

**Authors:** Aline Méndez-Rodríguez, Federica Rossetto, Heliot Zarza, Ricardo López-Wilchis, H. Christoph Liedtke, Juan E. Echevarria, Javier Juste

**Affiliations:** a Doctorado en Ciencias Biológicas y de la Salud, Universidad Autónoma Metropolitana. Ciudad de México, Mexico; b Tecnológico de Estudios Superiores de Huixquilucan, 52773, Estado de México, Mexico; c Instituto Pirenaico de Ecología, IPE-CSIC, Avda. Montañana 1005, Campus de Aula Dei, 50059, Zaragoza, Spain; d Departamento de Ciencias Ambientales, Universidad Autónoma Metropolitana-Lerma, Av. de las Garzas 10, Col. El Panteón, Lerma 52005, Mexico; e Departamento de Biología, Universidad Autónoma Metropolitana-Iztapalapa, Av. San Rafael Atlixco 186, Col. Vicentina, Iztapalapa 09340, Mexico; f Estación Biológica de Doñana, C.S.I.C., Avda. Américo Vespucio 26, 41092 Sevilla, Spain; g Centro Nacional de Microbiología, Instituto de Salud Carlos III, Madrid, Spain; h CIBER de Epidemiología y Salud Pública, CIBERESP, 28220 Madrid, Spain

**Keywords:** Chiroptera, Ecological traits, Genetic structure, Phylogenetic signal, Morphology, Systematic review, Viruses

## Abstract

Bats are recognized as natural reservoirs of a wide range of viruses. Understanding the factors that determine patterns of viral occurrence in reservoir species, particularly viral richness and viral detection frequency, is essential for explaining host–pathogen dynamics and anticipating public health risks. In this study, we evaluated the influence of life-history traits on viral frequency (defined as the proportion of positive detections across studies) in European bats for six important viral groups (*Adenoviridae, Herpesviridae, Papillomaviridae, Filoviridae, Coronaviridae,* and the genus *Lyssavirus* within *Rhabdoviridae*) through a systematic review, identifying 7,037 publications, of which 155 met the inclusion criteria. These studies focused primarily on three species: *Cnephaeus serotinus, Myotis daubentonii*, and *Pipistrellus pipistrellus*.

We detected a phylogenetic signal in viral frequency only for the family *Adenoviridae* and genus *Betacoronavirus*, suggesting that these viral groups have a particular evolutionary affinity with their hosts. Viral frequency was associated with bats’ environmental breadth and body size, although these relationships were not consistent across all viral groups. Instead, each viral group was associated with a distinct set of host life-history traits, reflecting -most probably- differences in their ecology and evolutionary history. Overall, our findings demonstrate that the ecological drivers of viral frequency are virus-specific rather than universal.

## Highlights

• Systematic review of viruses found so far in European bats (155 studies analyzed).
• Host body size and environmental breadth are common key predictors of viral frequency.
• Phylogenetic signal was detected for the virus families *Adenoviridae* and *Betacoronavirus*.
• Viral families show distinct associations with host life history.

## Graphical abstract

**Figure:**
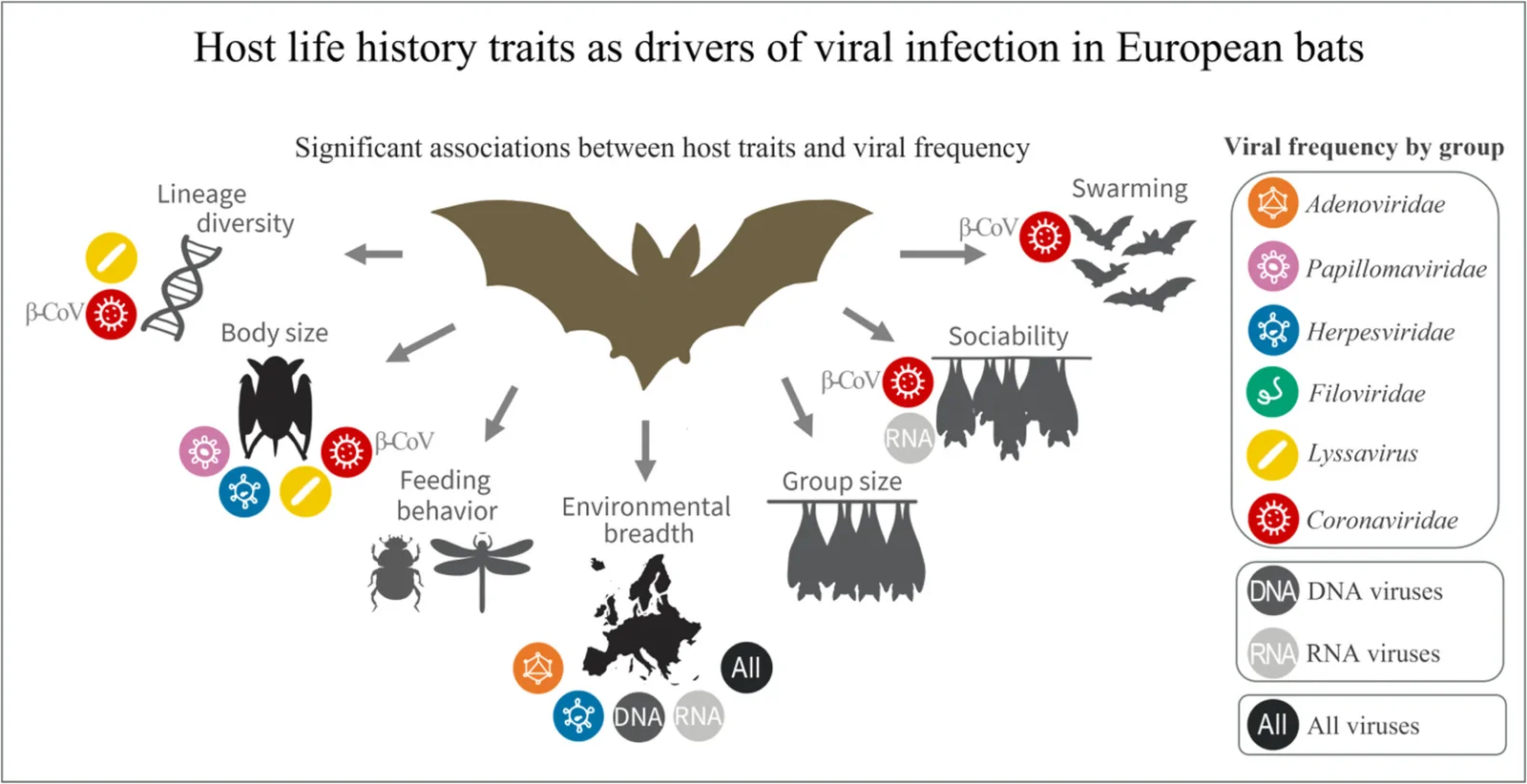

## Glossary

**SECIHTI**: Secretaría de Ciencia, Humanidades, Tecnología e Innovación
**DNA**: Deoxyribonucleic acid
**EBLV**: European Bat Lyssavirus
**EBLV-1**: European Bat Lyssavirus type 1
**ELISA**: Enzyme-Linked Immunosorbent Assay
**FAT**: Fluorescent Antibody Test
**FAVN**: Fluorescent Antibody Virus Neutralization test
**FIIT**: Fluorescent Inhibition Infection Test
**IFT**: Indirect Fluorescent Antibody Test
**IHC**: Immunohistochemistry
**IUCN**: International Union for the Conservation of Nature
**LLOV**: *Lloviu cuevavirus*
**mFAVN**: Modified Fluorescent Antibody Virus Neutralization test
**MERS-CoV**: Middle East Respiratory Syndrome Coronavirus
**NGS**: Next-Generation Sequencing
**PCR**: Polymerase Chain Reaction
**PRISMA**: Preferred Reporting Items for Systematic Reviews and Meta-Analyses
**RFFIT**: Rapid Fluorescent Focus Inhibition Test
**RNA**: Ribonucleic acid
**RTCIT**: Rapid Tissue Culture Infection Test
**SARS-CoV**: Severe Acute Respiratory Syndrome Coronavirus
**SARS-CoV-2**: Severe Acute Respiratory Syndrome Coronavirus 2

## 1 Introduction

Among mammals, bats stand out for their remarkable range of morphological, ecological, and behavioral characteristics (Simmons, 2003). Bats are also recognized as reservoirs of different viruses, some of which are of public health concern directly, such as the Marburg virus (Amman et al., 2020), Hendra virus (Field et al., 2011), Nipah virus (Epstein et al., 2020), and most lyssaviruses (Fooks et al., 2014), Although these viruses rarely manifest as clinical diseases in bats (O’Shea et al., 2014), they can impact the health of other species (Calisher et al., 2006; Letko et al., 2020). Bats are also considered the reservoirs of the recent ancestors of the Middle East Respiratory Syndrome Coronavirus (MERS-CoV) (Raj et al., 2014), Severe Acute Respiratory Syndrome Coronavirus (SARS-CoV) (Ge et al., 2013), and Severe Acute Respiratory Syndrome Coronavirus 2 (SARS-CoV-2), as well as likely reservoirs of Ebola viruses (Beltz, 2018; Pekar et al., 2025).

The high viral richness observed in bats may reflect the exceptional taxonomic diversity of the order Chiroptera (Mollentze and Streicker, 2020). However, this interpretation is still being debated. Several studies suggest that the viral richness in bats is unusually high and cannot be explained solely by their diversification (Calisher et al., 2006). It has also been linked to the unique evolutionary and ecological traits of this group of mammals (Luis et al., 2013). These traits include the capacity for flight and long-distance migration in some species, which facilitates viral dispersal; exceptional longevity relative to body size, which allows prolonged viral persistence and adaptation; gregarious behavior (ranging from a few individuals to millions), which increases contact and transmission opportunities; and physiological adaptations such as hibernation and daily torpor. In addition, bats possess an immune system capable of mounting a robust antiviral cytokine response while modulating the pro-inflammatory response, which may promote long-term host–virus coexistence (Baker et al., 2013; Calisher et al., 2006; O’Shea et al., 2014; Smith and Wang, 2013).

It has been proposed that certain ecological and life-history traits of the host are important determinants of viral circulation in bat populations because they influence host contact rates, exposure, transmission opportunities, and the persistence of infections (Han et al., 2016; Niu and McKee, 2025). Comparative studies have therefore investigated whether these traits (geographic distribution, longevity, body mass, reproductive frequency, litter size, genetic structure, colony size, sympatry with other species) explain interspecific differences in bat–virus associations, most commonly using viral richness as the response variable (Guy et al., 2019, 2020; Rossetto et al., 2020), particularly for viruses with zoonotic potential (Webber et al., 2017).

Traits that promote inter- and intraspecific contact, such as polygynous mating systems or high levels of sympatry, are expected to increase opportunities for pathogen transmission and, consequently, virus detection. Comparative studies have evaluated the relationship between these traits and bat–virus associations, although most have used viral richness as the response variable. Their conclusions remain inconsistent, with either positive (Han et al., 2016; Webber et al., 2017), negative (Rossetto et al., 2020), and non-significant (Guy et al., 2020; Lindenfors et al., 2007; Luis et al., 2013) associations reported. A similar lack of consensus is observed for other traits, such as colony size, body size, body mass, and geographic range (Guy et al., 2019, 2020; Lindenfors et al., 2007; Luis et al., 2013; Maganga et al., 2014; Turmelle and Olival, 2009; Webber et al., 2017).

These inconsistencies may partly reflect differences in the datasets analyzed, including the taxonomic and geographic scope (Maganga et al., 2014; Webber et al., 2017), the viral groups considered, as many studies focused primarily on zoonotic viruses (Guy et al., 2020; Han et al., 2016; Webber et al., 2017), and variation in sampling effort. Furthermore, many comparative studies pool viruses from different viral families into a single response variable (Lindenfors et al., 2007; Maganga et al., 2014; Turmelle and Olival, 2009), without considering that different viral families may respond differently to the ecological traits of their host. For example, phylogenetic component, sex, and mating strategy were identified as key factors for adenovirus in Spain (Rossetto et al., 2020), whereas filovirus presence was positively associated with colony size, degree of sympatry, and offspring number (Han et al., 2016).

Although viral richness and viral infection frequency are related aspects of host–virus interactions, they describe different biological processes. Viral richness refers to the number of distinct viruses associated with a host species. In contrast, viral frequency reflects the proportion of sampled individuals in which a virus has been detected. It is therefore influenced by epidemiological processes such as exposure, transmission dynamics, duration of infection, and sampling effort (Rothman et al., 2008). Consequently, analyses of viral frequency complement viral richness-based studies by providing insight into the ecological and epidemiological processes associated with virus detection across host species.

Given the scarcity of comparative analyses focusing on viral frequency across bat species, this review examines how characteristics of bats, such as their evolutionary history (lineage diversity), morphology (body size, length from canines to third molar and wing loading), and ecological traits (feeding behavior, group size, roost sharing and mating systems), influence viral frequencies, defined as the proportion of positive detection reported across studies. The study focuses on six viral groups (*Adenoviridae, Herpesviridae, Papillomaviridae, Filoviridae, Coronaviridae,* and the genus *Lyssavirus* within *Rhabdoviridae*). These viral groups were selected because they are among the most extensively studied viruses in bats, ensuring sufficient data availability for comparative analyses, and because they include both DNA- and RNA-based viruses with diverse transmission routes and host specificities (Wang and Cowled, 2015). Additionally, these groups have been frequently detected in various bat species and geographic regions, allowing robust estimation of viral frequencies.

To facilitate pattern identification and reduce the risk of overlooking relevant patterns, this review focuses on the entire European bat community, as Europe is one of the best-studied regions for bat-associated viruses (Dundarova et al., 2023; Hemnani et al., 2024; Méndez-Rodríguez et al., 2024). As a first step, we characterize research trends, including temporal trends in studies and the viral groups and bat species most frequently investigated. This overview describes the current research picture on bat viruses in Europe. We then evaluate how host traits influence viral frequencies across species.

Virus detection frequency is expected to depend on epidemiological processes such as host susceptibility, duration of infection, opportunities for transmission, persistence within populations, and the probability of exposure. We hypothesize that host traits that facilitate contact among conspecifics and between species influence viral frequency by altering opportunities for virus exposure and transmission. Furthermore, we hypothesize that species inhabiting a broader range of environments will exhibit greater viral frequency due to their exposure to a wider variety of ecological conditions and viral communities.

## 2 Materials and methods

### 2.1 Literature search and selection criteria

Following a PRISMA (Preferred Reporting Items for Systematic Reviews and Meta-Analyses) systematic review protocol (Moher et al., 2009; Page et al., 2021), we gathered information on the presence of viruses in the European bats. The search was conducted using Scopus and PubMed, using the following keywords: [bat AND (adenovirus OR *Adenoviridae*)], [bat AND (papillomavirus OR *Papillomaviridae*)], [bat AND (herpesvirus OR *Herpesviridae*)], [bat AND (filovirus OR *Filoviridae*)], [bat AND (rhabdovirus OR *Rhabdoviridae* OR *Lyssavirus*)] and [bat AND (coronavirus OR *Coronaviridae*)] and the database of bat-associated viruses (DBatVir, accessed January 1, 2024). Additionally, we reviewed the references included in the selected papers to identify additional relevant studies.

This comprehensive search included all studies published up to December 2023. Data were restricted to European bats, and geographical limits were defined according to Hackländer and Zachos (2023). Nomenclature followed Simmons and Cirranello (2025). For each species, we accounted for taxonomic changes over time, incorporating studies that used previous or synonymized species names. Species whose distribution was restricted to the Macaronesian biogeographic region (e.g., *Nyctalus azoreum, Pipistrellus maderensis, Plecotus gaisleri, Plecotus teneriffae*) or is marginal in continental Europe (e.g., *Pipistrellus hanaki, Plecotus gaisleri, Cnephaeus* (=*Eptesicus*) *anatolicus*) were excluded from the analysis. These species were excluded from statistical analyses due to their geographically peripheral distribution and the absence of virological data from Europe in the available literature. Their exclusion allowed us to focus on bat species with broader geographic distributions and sufficient data coverage, thereby reducing bias associated with data sparsity and ensuring more robust, comparable, and interpretable patterns of viral occurrence across European bats. Studies published in non-indexed journals, experimental studies, and review articles were excluded from the initial set of selected articles. Additionally, studies in which bats were not identified at the species level, as well as those based on pooled samples from multiple bat species, were also excluded.

For each selected article, two independent investigators (A.M.-R. and J.J.) screened the databases and extracted the relevant information. Discrepancies regarding article inclusion were resolved by consensus between the authors. The following information was recorded for each study: bat species; sampling location and year; number of individuals analyzed; number of positive individuals; total number of individuals sampled for each virus (when available); and detection methods used.

### 2.2 Database construction

Based on this information, two databases were constructed. The first one, hereafter referred to as the Viral Occurrence Database, recorded viral detection at the bat species–study level (i.e., for each bat species within each article). For each virus group, the detection status for each bat species in each study was classified as positive if the virus was detected, negative if it was tested but not detected, and not studied if it was not assessed. This classification was applied to the following five viral families: *Adenoviridae, Papillomaviridae, Herpesviridae, Filoviridae, Coronaviridae,* as well as the genus *Lyssavirus* (*Rhabdoviridae*). In this case, the grouping could not be maintained at the family level, since the vast majority of available information (over 96% of the studies) related only to *Lyssavirus* within the *Rhabdoviridae* family. This first database was used only to analyze research trends in viral detection.

The second database, hereafter referred to as the Viral Frequency Database, was constructed to estimate viral frequencies and evaluate the influence of life-history traits on this metric. This database included only records reporting both the number of positive individuals and the total number of bats sampled. This allowed estimating ‘viral frequency’, calculated as the number of positive bats divided by the total number of individuals sampled. Although this measure is formally equivalent to a proportion, we refer to it as viral frequency rather than prevalence because prevalence is defined as the proportion of infected individuals within a population at a given point in time or over a specified period, under a standardized sampling framework (Rothman et al., 2008). In contrast, our metric is derived from heterogeneous data sources with variable sampling effort and study design, and therefore does not represent a standardized epidemiological estimate. A study-level approach was not pursued because key variables such as sampling locality, sampling period, and diagnostic methodology were not consistently reported across publications. The viral grouping included the five families *Adenoviridae, Papillomaviridae, Herpesviridae, Filoviridae, Coronaviridae*, as well as the genus *Lyssavirus* within *Rhabdoviridae*. In contrast to the other viral families, most studies on *Coronaviridae* report viral identification at the genus level (*Alphacoronavirus* and *Betacoronavirus*), allowing us to analyze these groups independently without substantially reducing the number of available records. For the remaining viral families, finer taxonomic subdivision could not be applied because most studies do not consistently report viruses at lower taxonomic levels. Therefore, it would have considerably reduced the sample size and the statistical power of the analyses.

Finally, in the Viral Frequency Database, sufficient and consistent information was available only for *Lyssavirus* to allow further subdivision by the diagnostic method used to detect rabies. This reflects both the greater availability of data and the more extensive development of diagnostic approaches for *Lyssavirus* compared to other viral groups. Such detail was not consistently reported for the remaining viruses, precluding a similar classification without substantially reducing the sample size. Three data subsets were therefore defined only for *Lyssavirus*: 1) Active infection: detection of viral nucleic acids using Polymerase Chain Reaction (PCR), Next-Generation Sequencing (NGS), antigens using Fluorescent Antibody Test (FAT), Immunohistochemistry (IHC), Enzyme-Linked Immunosorbent Assay (ELISA), Indirect Fluorescent Antibody Test (IFT), or viral isolation by Rapid Tissue Culture Infection Test (RTCIT). 2) Serological evidence: detection of antibodies as an indicator of past exposure, though not necessarily active infection. Diagnostic methods included Fluorescent Antibody Virus Neutralization (FAVN), a modified version of FAVN (mFAVN), Rapid Fluorescent Focus Inhibition Test (RFFIT), Fluorescent Inhibition Infection Test (FIIT), as well as ELISA, and IFT, and 3) Combined evidence: a group including studies reporting both active infection and serological data, as well as those in which the diagnostic method was not specified. The latter were grouped to retain available data while acknowledging limitations in methodological resolution. This distinction was considered biologically relevant because active infection and serological evidence provide complementary information about virus circulation in host populations. Separating these categories allowed us to assess whether patterns observed for current infection were consistent with those observed for previous exposure.

In a subsequent design, viruses were grouped according to their nucleic acid type (DNA or RNA) in order to explore possible patterns based on their molecular characteristics. Finally, all data were combined into a single category (all viruses) and analyzed together to identify overall frequency patterns.

During data compilation, articles using next-generation sequencing (NGS) were excluded when samples were processed in pools without distinguishing either the species identity or the number of individuals contributing to each pool. Under these conditions, viral frequency could not be estimated. Studies in which samples were processed collectively, even when focusing on a single species, prevented the determination of the number of positive and negative individuals. Therefore, these studies were excluded from the Viral Frequency Database but retained in the Viral Occurrence Database.

### 2.3 Selection of variables

Ten species-level traits were compiled from the bibliography on European bats. All these traits have previously been found to be important predictors of richness, diversity and prevalence in mammals, particularly in bats (Guy et al., 2020, 2019; Han et al., 2016; Maganga et al., 2014; Rossetto et al., 2020; Webber et al., 2017). The traits were selected to correspond to emergent properties -defined as characteristics that cannot be observed at the individual level but arise from the interactions among individuals or species (Nielsen and Müller, 2000). Considering these properties enabled a comprehensive assessment of the ecological mechanisms occurring within populations and communities that may influence viral diversity.

Trait selection was guided by their biological relevance to bat host ecology and their potential influence on viral dynamics, particularly through effects on contact rates, pathogen exposure, and movement patterns. To ensure comparability among species, only traits for which data were available for all bat species included in the analyses were retained. When multiple values were reported in the literature for a given trait, representative estimates were selected according to the nature of each variable, as described in Table S1.

1.- *Lineage diversity*: Number of well-supported genetic lineages in the molecular evolutionary reconstructions using either nuclear or mitochondrial DNA markers found in the bibliography for each species; 2.- *Body size*: Average forearm length (mm) reported for the species; 3.- *Length from canines to third molar* (*CM3*): Distance (mm) from the anterior edge of the canine to the posterior edge of the third molar. This craniodental measurement characterizes skull morphology. Cranial and dental morphology have been shown to be closely associated with dietary specialization in bats (Dumont, 2007); 4.- *Wing loading*: Defined as the body mass divided by total wing area (g/dm²), is reflecting flight performance and maneuverability, and is closely associated with the bat’s movement ecology and dispersal capacity (Luo et al., 2019; Norberg and Rayner, 1987); 5.- *Feeding behavior*: Number of orders found as primary food items in diet studies of each species; 6.- *Environmental breadth*: Number of biogeographic regions (Alpine, Atlantic, Black Sea, Boreal, Continental, Macaronesian, Mediterranean, Pannonian and Steppic region) where the species is found; 7.- *Group* size: Maximum number of individuals reported for the species in summer roosts. This variable was used as a proxy for aggregation and potential contact rates during the reproductive season. Summer colonies correspond to ‘maternity colonies’ and were selected because they are more consistently reported across studies and represent periods of high social interaction, whereas winter aggregations correspond to a reduced metabolism period (hibernation), less individual contacts and with a greater variability across species (Martínková et al., 2020; Webber and Willis, 2016); 8.- *Sociability*: Indicating the tendency of sharing summer roosts with other species, categorized into two groups: species that have never been observed sharing roosts with other species, and species that sometimes or always share roosts with other species; 9.- *Swarming*: Whether the species form multi-specific aggregations in particular places in autumn and exhibit swarming mating behavior or not; 10.- *Sampling effort*: Total number of individual bats sampled for each species and for each viral group analyzed (Table S2 and Table S3).

### 2.4 Phylogenetic signal

Phylogenetic non-independence means that observed relationships among the above traits in bat species may reflect shared evolutionary history and therefore need to be accounted for in comparative analyses. Since a fully resolved phylogeny for the 38 European bat species considered in this study was not available in the literature, a composite tree was assembled as a proxy. Topological relationships were established by combining published phylogenies (Juste et al., 2004; Mata et al., 2017; Mucedda et al., 2002; Shahabi et al., 2019), all of which report strong support for the inferred relationships. The relationships supported by these studies were combined into a single composite topology using the ‘ape’ package in R version 4.4.1 (Paradis et al., 2004). The rhinolophid clade (*Rhinolophus blasii, R. euryale, R. ferrumequinum, R. hipposideros*, and *R. mehelyi*) was designated as the outgroup, given that horseshoe bats belong to a different suborder within Chiroptera (Lei and Dong, 2016).

To estimate branch lengths for this constrained topology, mitochondrial sequences of cytochrome b (1140 base pairs) and NADH dehydrogenase 1 (665 base pairs) were obtained from GenBank. Most sequences corresponded to different individuals of the same species (Table S4). Each locus was aligned using the MUSCLE algorithm implemented in MEGA X (Kumar et al., 2018) and subsequently concatenated. The resulting alignment was analyzed in RAxML v8.2.12 (Stamatakis, 2014), using the composite topology as a topological constraint. Codon positions were treated as independent partitions, and the substitution model was set to GTRGAMMA.

The resulting additive tree was converted into an ultrametric tree using the *chronos()* function in the R package ‘*ape*’ (Paradis et al., 2004). Different clock models were tested, including the "correlated" and "relaxed" models, to account for alternative assumptions regarding rate variation among lineages. Both models resulted in similar likelihood scores (-33.89 and -33.11, respectively). Given their comparable performance, the "correlated" model was selected for the final analysis. The resulting tree is provided in Supplementary Material 1.

To estimate the phylogenetic signal underlying the frequency patterns of each virus across European bat species, we employed Pagel’s λ parameter estimated with the package ‘*phytools*’ (Revell, 2012). Pagel’s λ measures the extent to which variation in a trait is associated with phylogenetic relatedness by scaling the expected covariance among species, where λ = 0 indicates that related species do not share similar values for the trait (viral frequency) and λ = 1 indicates that trait variation is consistent with a Brownian motion model of evolution, with closely related species tending to have similar values (Pagel, 1999).

### 2.5 Multicollinearity and linear models

To examine the relationship between viral frequency and life history traits in European bats, viral frequency was used as the dependent variable, and ten species-level traits were included as independent predictors (*Lineage diversity, Body size, CM3, Wing loading, Feeding behavior, Environmental breadth, Group size, Sociability, Swarming,* and *Sampling effort*). Prior to model fitting, continuous predictors were log-transformed to improve distributional symmetry and reduce heteroscedasticity.

To assess potential non-independence among life-history traits (multicollinearity), we constructed pairwise correlation matrices separately for each viral family, genus, and viral nucleic acid type, as the set of bat species included varied among virus groups due to differences in data availability (i.e., not all species had been studied for all viruses). Correlations were estimated using the heterogeneous correlation model implemented via the *hetcor()* function of the *‘polycor’* R package (Fox and Fox, 2016). This approach estimates Pearson correlations for continuous variables, polyserial correlations for combinations of continuous and categorical variables, and polychoric correlations for categorical variables, thus providing appropriate correlation estimates for heterogeneous datasets. Variables showing strong collinearity (|r| > 0.7) were not included simultaneously in subsequent models to avoid redundancy and instability in parameter estimates (Dormann et al., 2013).

Once independence between variables was confirmed (see Results), we evaluated the association between viral frequency in bats and life-history traits using phylogenetic linear models implemented with the *phylolm()* function in the ‘*phylolm*’ R package (Tung Ho and Ané, 2014). Viral frequency was used as the response variable, and life-history traits were included as explanatory variables. Separate models were fitted for each viral group because the set of bat species with available data differed among virus groups. The species-level phylogeny was included in each model, assuming a Brownian motion model of trait evolution (Symonds and Blomberg, 2014). Separate analyses were conducted for each virus group, including adeno-, papilloma-, herpes-, filo-, rhabdo-, *Lyssa*-, corona-, *Alphacorona*-, *Betacorona*-, DNA viruses, RNA viruses, and all viruses.

Compliance with model assumptions was assessed by testing the normality of model residuals using the Shapiro-Wilk test with the s*hapiro.test()* function in the R package *‘stats’* (R Core Team, 2024). When deviations from normality were detected, response variables were log₁₀ transformed, and the model was refitted. If normality was not adequately improved after log-transformation, a Box–Cox transformation was applied using the *boxcox()* function from the ‘*MASS*’ R package (Venables and Ripley, 2002). For each model, we extracted the estimated regression coefficients (β), standard errors, t-values, degrees of freedom, and p-values from the *phylolm()* output. We also calculated 95% confidence intervals for the estimated coefficients. Positive coefficients indicate positive associations between a trait and viral frequency, whereas negative coefficients indicate negative associations. All analyses were performed in R version 4.4.1 (R Core Team, 2024), and scripts are available upon request.

## 3 Results

The PRISMA-based literature review identified a total of 7,037 relevant publications. After reviewing the references cited in the first selected studies, an additional 156 publications were included. Following the application of the exclusion criteria (removal of duplicates, non-observational experimental studies, review articles, and reports based on samples from captive bats), a total of 155 publications were selected for analysis (Fig. S1 and Table S5). Most of the excluded articles were experimental studies, did not include viral identification in bats, or focused on bat species or geographic regions not considered in this review.

The selected publications compile data on bats from 25 European countries. Research effort was unevenly distributed across the continent, with a small number of countries accounting for most of the studies, whereas many other countries have few or no records. The number of studies on viruses in European bats has increased over time, with a marked rise in recent years. The highest numbers of publications were recorded in 2014 and 2022, with 11 studies each (Fig. S2).

The species *Cnephaeus* (=*Eptesicus*) *serotinus* (82 publications), *Myotis daubentonii* (70), and *Pipistrellus pipistrellus* (55) were the most frequently studied bats, whereas *M. crypticus* (2 publications), *Rhinolophus blasii* (2), and *M. punicus* (1) were the least represented in the scientific literature (Fig. S3). On the other hand, 66.45% of the publications focused on the genus *Lyssavirus*, and on the bats *C. serotinus* (59 publications) or *M. daubentonii* (47 publications). At the same time, *Papillomaviridae* was the least researched group, with only 6.45% of the publications reviewed related to this viral family (Fig. S4).

*Coronaviridae* stood out as the group with the widest host range, as they were detected in 32 of the 37 bat species studied (86.5%), covering the four bat families present in Europe. They were followed by *Herpesviridae* (27 bat species and 73%), *Adenoviridae* (25 bats and 67.6%), *Lyssavirus* (23 bats and 62%), *Papillomaviridae* (5 bats: *Hypsugo savii, C. serotinus, C. isabellinus, M. brandtii*, and *R. ferrumequinum*, representing 13% of the European bats), and *Filoviridae,* which were found in *Miniopterus schreibersii*. The latter two viral families not only exhibited the lowest presence among the possible host species but were also the least studied.

No bat species tested positive for all six viral groups (Fig. 1). However, *P. pipistrellus, H. savii, C. serotinus, M. schreibersii*, and *R. ferrumequinum* tested positive for five of the six viral groups. By contrast, no viruses were detected in *Plecotus macrobullaris*. This species was analyzed using Next-Generation Sequencing (NGS), as well as Polymerase Chain Reaction (PCR) targeting *Coronaviridae* and *Lyssavirus*, and *Lyssavirus* antigen detection. Nevertheless, due to the small number of individuals sampled (n ≈ 9), one should be prudent in concluding viral absence in this species.

**Fig. 1:**
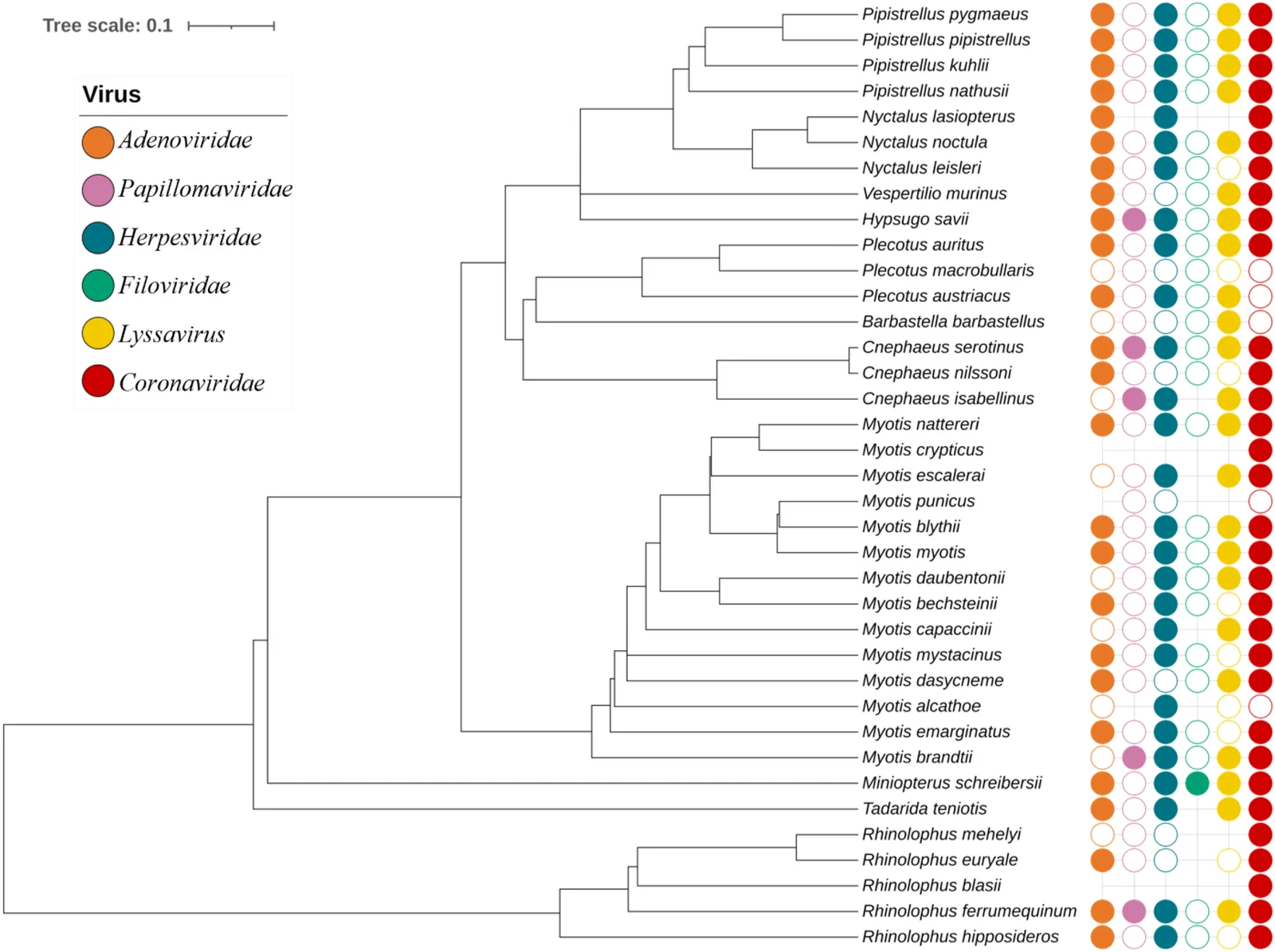
Viral detection of the six selected viral families shown in relation to the inferred evolutionary relationships among the European bats included in this study. The topology was assembled from previously published, well-supported phylogenies (Juste et al., 2004; Mata et al., 2017; Mucedda et al., 2002; Shahabi et al., 2019); length of the branches was estimated in RAxML v8.2.12 (Stamatakis, 2014), using the composite topology as a topological constraint. Branch support values are not reported because the tree was not inferred de novo. Filled circles represent positive viral detections, empty circles (outline only) indicate negative results, and the absence of a symbol denotes that the virus was not tested in the corresponding bat species. Viral detection data correspond to the Viral Occurrence Database described in the Materials and Methods.

### 3.1 Selection of variables

Correlation coefficients were calculated for all pairwise combinations of the ten traits: *lineage diversity, body size, CM3, wing loading, feeding behavior, environmental breadth, group size, sociability, swarming*, and *sampling effort*. When collinearity was detected, variable retention was guided by biological interpretability and by reducing redundancy among predictors.

*Lineage diversity, body size, feeding behavior, environmental breadth, group size, sociability,* and *swarming* showed pairwise correlations below the collinearity threshold (|r| < 0.7), supporting their inclusion in the comparative analyses (Fig. S5). In contrast, *CM3, wing loading,* and *sampling effort* showed correlations exceeding this threshold with other traits in multiple viral groupings, indicating redundancy.

*Wing loading* and *CM3* were strongly correlated with each other and with *body size*, indicating that these variables capture overlapping aspects of morphological variation. Among these correlated traits, *body size* was retained because it is a widely used, integrative descriptor of bat morphology, whereas *wing loading* and *CM3* were excluded. *Sampling effort* was excluded because it reflects study design rather than an intrinsic species trait and was already accounted for in estimating viral frequency. Consequently, seven traits were retained for subsequent analyses.

### 3.2 Viral frequency and phylogenetic signal

Viral frequency varied considerably both between bat species and between the different types of viruses analyzed (Table S6). Only *Adenoviridae* and *Betacoronavirus* exhibited a significant phylogenetic signal in viral frequency among bat species (λ = 0.8912, p = 0.0011 and λ = 1.0622, p = 0.0114), while the remaining viruses did not show evidence of phylogenetic dependence, whether analyzed individually or grouped into categories (Table S7).

After correcting for phylogenetic non-independence, the life history traits that significantly explained viral frequency varied by viral grouping (Table 1 and Table S7). After transformation, model residuals met the assumption of normality across viral groups (Table 1).

**Table 1:** Bat life-history traits are significantly associated with viral frequency (p < 0.05) across viral groupings. For each association, effect sizes are reported as regression coefficients (β) with their corresponding 95% confidence intervals (CI_low–CI_high) and p-values. A ‘+’ indicates a positive association, whereas a ‘–’ indicates a negative association. The transformation applied to the response variable is indicated for each model. Full statistical results, including non-significant predictors, are provided in Table S7.

Virus	Data transformation	Traits with p<0.05
***Adenoviridae***	Log10	+ *Environmental breadth* β=8.5131 CI lower=1.6627 CI upper=15.3636 p=0.0220
***Papillomaviridae***	Log10	+ *Body* s*ize* β=42.1354 CI lower= 25.6129 CI upper=58.6579 p=4.174e-05
***Herpesviridae***	Log10	+ *Body size* β=33.4393 CI lower=16.7393CI upper=50.1393 p= 0.0005 *+ Environmental breadth* β=9.6008 CI lower=3.4445CI upper=15.7571 p=0.0051
***Lyssavirus***	Log10	+ *Lineage diversity* β=7.5003 CI lower=3.1134 CI upper=14.8873 p=0.0480 + *Body* s*ize* β=42.9924 CI lower=24.5192 CI upper=61.4656 p= 0.0001
***Coronaviridae***	Box-Cox	No significant associations
***Alphacoronavirus***	Log10	No significant associations
***Betacoronavirus***	Log10	+ *Lineage diversity* β=0.7552 CI lower=0.1307 CI upper=1.3798 p=0.0419 + *Body* s*ize* β=3.1006 CI lower=0.5892 CI upper=5.6120 p=0.0386 - *Sociability* β=-0.6559 CI lower=-1.0389 CI upper=-0.2729 p=0.0084 + *Swarming* β=0.6663 CI lower=0.1134 CI upper=1.2193 p=0.0424
**DNA viruses**	Log10	+ *Environmental breadth* β=5.1764 CI lower=1.1005 CI upper=9.2524 p=0.0193
**RNA viruses**	Log10	+ *Environmental breadth* β=5.1293 CI lower=2.2880 CI upper=7.9707 p=0.0013 + *Sociability* β=2.3321 CI lower=0.4435 CI upper=4.2207 p=0.0220
**All viruses**	Log10	+ *Environmental breadth* β=5.7175 CI lower=3.3404 CI upper=8.0947 p=0.0001

For the family *Adenoviridae*, viral frequency was positively associated with *environmental breadth*, indicating that species occupying a broader range of ecological conditions tended to exhibit higher frequencies of this virus family. In the case of *Papillomaviridae*, viral frequency was positively associated with *body size*, indicating that viral frequency tends to be higher in larger bats (Table 1). Similarly, *Herpesviridae* showed positive associations with both *body size* and *environmental breadth* (Table 1).

For *Lyssavirus*, the diagnostic method used to detect infection (active infection, serological evidence, or combined evidence) did not influence the results on the selection of life history traits associated with viral frequency (Table S5). Because results were consistent across datasets, only the analyses based on combined evidence are presented in the main text, while the remaining results are provided as Supplementary Material. In this dataset, *Lyssavirus* viral frequency was positively associated with *lineage diversity* and *body size*.

No significant associations were found between viral frequency and the selected traits when considering the family *Coronaviridae* as a whole. However, when the analysis was broken down by genus, *Betacoronavirus* showed positive associations with *lineage diversity, body size*, and *swarming* behavior, and a negative association with *sociability*. In contrast, *Alphacoronavirus* did not show significant associations with any evaluated traits. Finally, Filoviruses had to be excluded from the analysis because they did not meet the models’ assumptions of normality, likely due to the low sample size (Fig. 2).

**Fig. 2:**
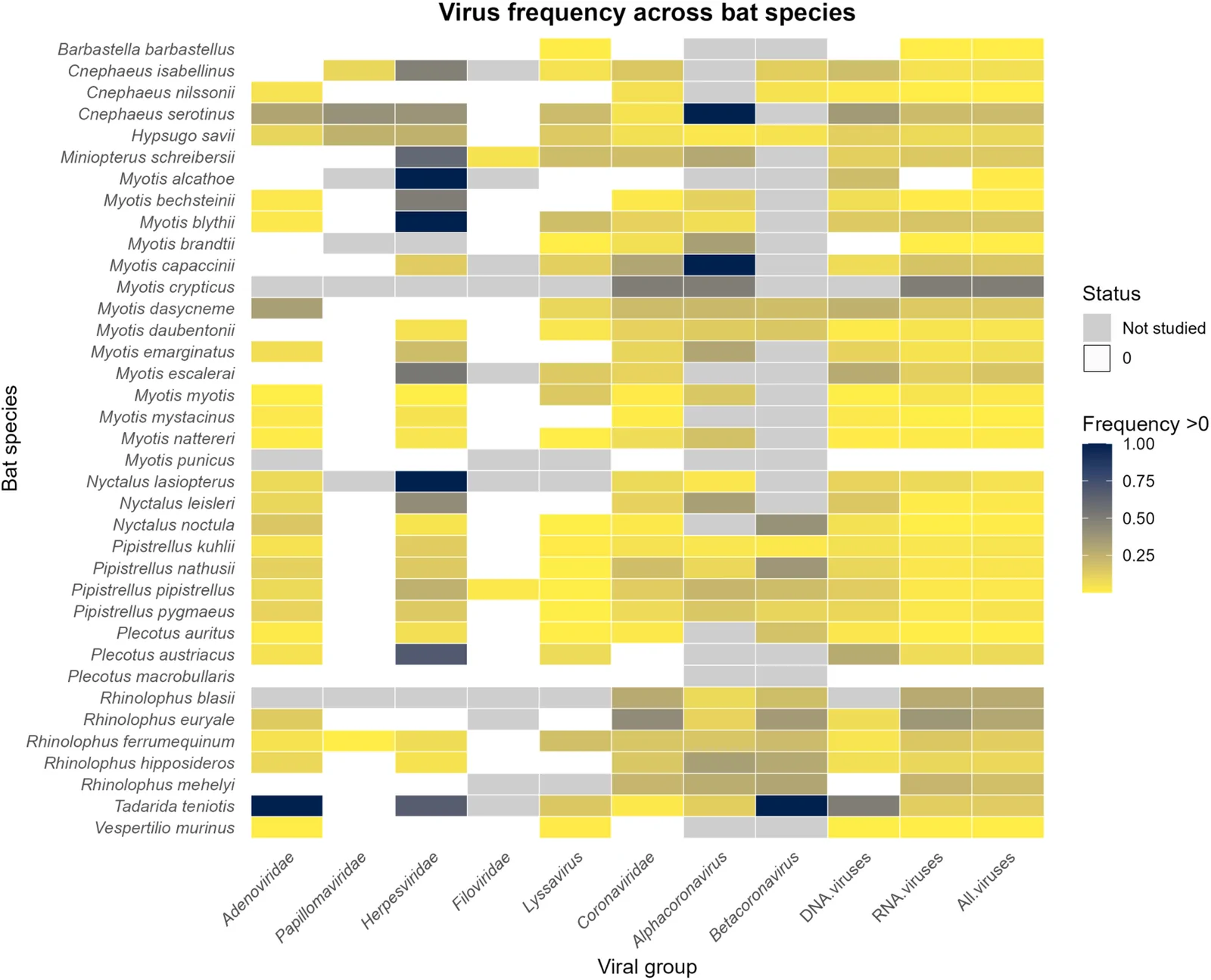
Viral frequency of *Adenoviridae, Papillomaviridae, Herpesviridae, Filoviridae, Lyssavirus, Coronaviridae, Alphacoronavirus, Betacoronavirus*, viruses grouped by nucleic acid type (DNA and RNA), and all viruses, across European bat species. Each cell represents the relative frequency for a given species–virus combination. Gray cells indicate combinations that have not been studied, whereas white cells indicate zero frequency. Viral detection data correspond to the Viral Frequency Database described in the Materials and Methods. The number of individuals sampled and the number of positive detections for each bat species and viral group used to estimate viral frequency are provided in Table S6.

At a broader taxonomic scale, viral frequency was positively associated with bats’ *environmental breadth* for DNA viruses and RNA viruses; the latter also showed a positive association with interspecific contacts (*sociability*). *Environmental breadth* was likewise associated with viral frequency in the combined analysis of all viruses.

In summary, associations between host traits and viral frequency varied across viral groups. Whereas *environmental breadth* and *body size* were associated with viral frequency across several analyses, *lineage diversity* and *sociability* were associated with viral frequency only in a few analyses. *Swarming* exhibited associations only in specific viral groups.

## 4 Discussion

Bats are recognized as natural reservoirs of a wide range of viruses that are thought to rarely cause clinical disease in their hosts (O’Shea et al., 2014), although recent evidence suggests that their responses to novel infections may be more similar to those of other mammals than previously assumed (Farrell et al., 2025). These observations have stimulated considerable interest in understanding the ecological and evolutionary factors that shape bat–virus associations. Most comparative studies have used viral richness as the response variable to investigate bat–virus associations.

In contrast, our study focuses on evaluating viral frequency, a complementary metric that reflects the proportion of sampled individuals in which viruses are detected and is therefore more closely related to epidemiological processes such as exposure, transmission opportunities, and infection persistence.

By analyzing viral frequency separately for six viral groups, rather than pooling all viruses into a single response variable, our study explores whether different viral families are associated with distinct host life-history traits. We additionally examined viruses according to their genome type (DNA versus RNA). However, it is important to note that these groupings are based on a limited subset of viral families (*Adenoviridae, Papillomaviridae*, and *Herpesviridae* for DNA viruses; *Filoviridae, Lyssavirus*, and *Coronaviridae* for RNA viruses). Consequently, the observed differences between DNA and RNA viruses should be interpreted with caution.

### 4.1 Research trends

The literature review highlights marked differences in the research effort devoted to different viral groups. These differences are important because they influence the amount of information available for estimating viral frequency and interpreting host–virus associations.

The analyses reveal a marked bias in virus groups that have received unequal scientific attention, with DNA viruses generally less studied than RNA viruses, likely due to their perceived lower zoonotic risk. Among the first, adenoviruses and herpesviruses have been widely studied and detected in 27 and 25 of the 37 European bat species, respectively, showing high diversity even within single host species (Pozo et al., 2016; Vidovszky et al., 2015). Although some groups within these virus families have traditionally been considered host-specific (Pozo et al., 2016; Rossetto et al., 2020), the detection of closely related viral species across bat species suggests more complex evolutionary dynamics (Méndez-Rodríguez et al., 2024; Vidovszky et al., 2015). Papillomaviruses have been reported in only a limited number of European bats, with their detection largely dependent on next-generation sequencing due to the scarcity of bat-specific primers, which likely underestimates their occurrence across European bats.

Among RNA viruses, the *Lloviu cuevavirus* (LLOV) is the sole filovirus identified in Europe to date, and it seems to be confined to the bat *M. schreibersii* in Spain, Italy, and Hungary (De Arellano et al., 2019; Kemenesi et al., 2018, 2022; Negredo et al., 2011; Tóth et al., 2023). This species specificity hinders the assessment of associations with host traits and viral frequency. Additional research is needed to better understand the ecology and evolutionary dynamics of LLOV, particularly given its zoonotic potential has been demonstrated in vitro (Hume et al., 2022; Kemenesi et al., 2022).

In contrast, *Lyssavirus hamburg* and *Lyssavirus helsinki* (EBLVI and EBLV2) have been the focus of extensive research due to their zoonotic relevance, largely through national surveillance programs across Europe. The use of different detection methods, including PCR, antigen-based assays, and serological testing, has facilitated a better understanding of viral circulation, particularly in the bats *C. serotinus* and *C. isabellinus*, recognized as primary hosts of the EBLV-1 (Mingo-Casas et al., 2017).

Coronaviruses have also been the focus of considerable attention following recent global health emergencies. They have been detected in most European bat species and show a wide range of detection frequencies (0.007 to 0.430), suggesting substantial heterogeneity among host species. This variability likely reflects differences in infection dynamics, host susceptibility, and exposure. Their presence on a variety of bat species, and at times in high abundance, supports a long-standing evolutionary association between bats and coronaviruses (Woo et al., 2023).

### 4.2 Selected life history traits

Our analyses identified significant associations between several life-history traits and viral frequency, although these relationships differed among viral groups and no single trait consistently explained virus detection across all analyses.

Pointing to our original hypothesis. *Environmental breadth* was the life-history trait most consistently associated with viral frequency, showing positive relationships with *Adenoviridae, Herpesviridae*, the grouped DNA viruses, RNA viruses, and the complete dataset. This association suggests that, as predicted, species with higher ecological plasticity, and therefore occupying a broader range of environments, show higher viral frequency, probably because they encounter greater diversity of flora and fauna and have more opportunities for virus exposure and maintenance across populations. In contrast, traits related to intra- and interspecific contact among bats were primarily associated with *Betacoronavirus* frequency, suggesting that host contact patterns may be particularly important for the transmission dynamics of this particular group of viruses but do not represent an important mechanism for other bat-associated viruses.

#### 4.2.1 Lineage diversity

Our analyses identified a positive association between *lineage diversity* and the frequency of *Lyssavirus* and *Betacoronavirus* detection. *Lineage diversity*, defined as the number of well-supported lineages identified from mitochondrial and nuclear phylogenetic evidence, which reflect historical genetic, structuring, and complexity of a species. Consequently, species with more lineages may have experienced more complex demographic histories, potentially influencing host–virus interactions through distinct biological mechanisms.

For *Lyssavirus*, no significant phylogenetic signal was detected in our analyses at the genus level. However, this result should be interpreted cautiously because several *Lyssavirus* species and lineages exhibit preferential associations with particular bat species (Davis et al., 2005; Franka et al., 2006; Velasco-Villa et al., 2006; McElhinney et al., 2013; Wang and Cowled, 2015). In the present study, *Lyssavirus* detections were analyzed at the genus level because many publications did not identify to species. Restricting the analyses to species-level identifications would have substantially reduced the number of available records and the statistical power of the analyses.

Phylogeographic studies further suggest that viral circulation varies among *Lyssavirus* species according to the historical structure and connectivity of their bat hosts. For example, Davis et al. (2005) found that EBLV-1a exhibited relatively weak spatial structure, suggesting long-distance viral exchange maintained by connected or migratory bat populations, whereas EBLV-1b exhibited stronger geographic subdivision, suggesting reduced connectivity among host populations. Although these studies did not directly evaluate lineage diversity, they indicate that historical host population structure can shape viral circulation and maintenance over broad geographic scales. Although no significant phylogenetic signal was detected when *Lyssavirus* was considered as a single viral group, *lineage diversity* was positively associated with its frequency. This suggests that *Lyssavirus* frequency may be related not only to a particular bat host species *per se*, but also to the degree of genetic diversification within species. Species comprising multiple genetic lineages represent a more spatially and genetically structured host system, providing multiple opportunities for local viral maintenance, diversification, and subsequent reintroduction among host groups. This interpretation is consistent with modeling studies showing that *Lyssavirus* persistence can depend on host movement, spatial structure, and the exchange of infected individuals among populations (Colombi et al., 2019). Importantly, *group size* was not associated with *Lyssavirus* frequency in our analyses, suggesting that the observed pattern is not simply driven by larger colonies or greater local host density.

In contrast, the association between *lineage diversity* and *Betacoronavirus* frequency seems to be more consistent with long-term evolutionary relationships between hosts and viruses. Previous studies have demonstrated strong host specificity and phylogenetic congruence among betacoronaviruses, suggesting that host evolutionary history contributes to shaping virus distribution (Razanajatovo et al., 2015).

Likewise, SARS-related betacoronaviruses have repeatedly been associated with bats of the genus *Rhinolophus* throughout Asia (Latinne et al., 2024; Luk et al., 2019). Consistent with these observations, *Betacoronavirus* also exhibited a significant phylogenetic signal in our analyses, supporting the hypothesis that host evolutionary history contributes to differences in viral frequency among European bats. Although the representation of *Rhinolophus* species in our dataset may have influenced this pattern (Table S3), betacoronaviruses were also detected in several other bat genera, indicating that the observed association is unlikely to be explained exclusively by these bats.

Overall, our findings suggest that *lineage diversity* may be associated with viral frequency through distinct mechanisms across viral groups. For Betacoronavirus, the association with *lineage diversity* and the significant phylogenetic signal are consistent with host evolutionary history and host–virus specificity. Whereas, in the case of *Lyssavirus*, the positive association may reflect the capacity of genetically structured host species to provide multiple opportunities for viral maintenance and reintroduction across host groups.

#### 4.2.2 Body size

Forearm length, a standard measure of body size in bats, was positively associated with the frequency of papillomavirus, herpesvirus, *Lyssavirus*, and *Betacoronavirus*. Body size has consistently emerged as one of the host traits associated with patterns of viral diversity in bats and other mammals (see Castro et al., unpublished results; Guy et al., 2019; Lindenfors et al., 2007; Luis et al., 2013; Maganga et al., 2014). Although viral diversity in all these studies was primarily evaluated as viral richness rather than viral frequency, they collectively identify body size as an important ecological trait influencing host–virus associations.

One proposed explanation involves the unique physiology of powered flight. The "flight-as-fever" hypothesis suggests that the repeated elevations in body temperature experienced during flight have favored immune adaptations that promote viral tolerance and persistence in bats (O'Shea et al., 2014). Building on this framework, Castro et al. (unpublished results) proposed that differences in heat dissipation associated with body size may have contributed, over evolutionary time, to the greater viral richness observed in larger bat species. Although this hypothesis was developed to explain patterns of viral richness, the same physiological mechanisms may also contribute to the positive association between body size and viral frequency observed in our analyses.

#### 4.2.3 Feeding behavior

None of the virus groups showed a significant association between viral frequency and host diet diversity. This indicates that the feeding strategy was not associated with viral frequency in the virus groups analyzed. However, NGS-based studies on total viromes have shown that diet influences the overall viral composition detected in bats. In contrast, metagenomic studies have shown that diet strongly influences the overall viral composition of bat viromes through the ingestion of insect-, plant-, and avian-associated viruses (Cholleti et al., 2023; Huang et al., 2024; Li et al., 2010; Méndez-Rodríguez et al., 2026; Šimić et al., 2018).

These viruses differ from those evaluated in this study, which focused on viruses known to infect bats. Therefore, it is important to distinguish between viruses inherent to the host and those derived from the diet to accurately interpret viromic data.

#### 4.2.4 Environmental breadth

Our models indicate that bat species occupying a greater number of geographic regions exhibited higher viral frequencies of *Adenoviridae, Herpesviridae*, DNA viruses, RNA viruses, and all viruses combined. These findings suggest that species with broader geographic distributions experience greater opportunities for viral exposure and transmission across heterogeneous environments (Turmelle and Olival, 2009) and as a result, they show higher viral frequency.

Similar studies have consistently identified geographic distribution as an important host trait associated with parasite and viral diversity in mammals (Gregory, 1990; Kamiya et al., 2014; Lindenfors et al., 2007; Maganga et al., 2014; Nunn et al., 2003). Although these studies again evaluated viral diversity focusing primarily on richness rather than on viral frequency, they support the hypothesis that broader geographic distributions increase opportunities for host–virus contact and viral circulation, providing a plausible explanation for the positive associations observed in our analyses.

#### 4.2.5 *Sociability* and *Swarming*

Our initial hypotheses predicted that bat species sharing roosts with other species, or exhibiting swarming behavior, would be more exposed to viral transmission and maintenance events and therefore, would show higher viral diversity. However, we found that only the frequency of *Betacoronavirus* was positively associated with *swarming behavior* and negatively associated with interspecific roosting (*sociability*). While this pattern may seem contradictory, both traits represent distinct ecological processes with possibly different implications for virus transmission.

Swarming consists of temporary, high-density aggregations of individuals from multiple populations and species, during the mating season (Parsons et al., 2003), creating favorable conditions for the transmission of viruses through close contact, including respiratory or fecal–oral routes, such as *Betacoronavirus* (Beltz, 2018). These seasonal aggregations may therefore facilitate viral circulation among populations while maintaining transmission primarily within species. In contrast, interspecific roosting could increase opportunities for contact and exposure between bat species, but such exposure does not necessarily result in cross-species infection or sustained transmission. In general, bat species segregate spatially or temporally within shared roosts according to their ecological preferences (Kunz and Fenton, 2005). Besides, host-specific barriers, including receptor compatibility and immune differences associated with phylogenetic distance, may constrain the establishment of infection following exposure (Caraballo, 2022; Mingo-Casas et al., 2017). Thus, although interspecific roosting may facilitate exposure to viruses circulating in other bat species, the resulting increase in contact does not necessarily translate into higher viral frequency.

## 5 Conclusions and future directions

This study highlights important gaps in our understanding of viral diversity in European bats, largely resulting from uneven sampling across bat species and viral families. Some viral families (*Papillomaviridae* and *Filoviridae*) and bat species (e.g., *M. crypticus, P. macrobullaris,* and *R. mehelyi*) remain poorly represented, underscoring the need for broader geographic and taxonomic sampling efforts, along with non-targeted viral surveillance, to obtain a more comprehensive characterization of bat viromes.

By distinguishing viral frequency from viral richness, this study provides a complementary perspective on host–virus associations in European bats. Our results demonstrate that the ecological drivers of viral frequency differ among viral groups, indicating that host ecological traits do not exert uniform effects across bat-associated viruses. *Environmental breadth* showed the broadest associations with viral frequency, whereas *body size, lineage diversity, swarming* behavior, and *sociability* were associated with specific viral groups, reflecting differences in viral ecology, transmission, and evolutionary history.

These findings indicate that the processes shaping viral frequency are virus-dependent and should therefore be interpreted within the ecological and evolutionary context of each viral group rather than generalized across all bat viruses. We have to consider also that these patterns should be interpreted within the frame of the viral groups analyzed, which, despite including some of the most extensively studied bat-associated viruses, may not represent all DNA and RNA viruses.

## CRediT authorship contribution statement

**Aline Méndez-Rodríguez:** Writing – review & editing, Writing – original draft, Visualization, Methodology, Investigation, Formal analysis, Data curation. **Federica Rossetto:** Writing – review & editing, Validation, Software, Methodology, Formal analysis. **Heliot Zarza:** Writing – review & editing, Supervision. **Ricardo López-Wilchis:** Writing – review & editing, Supervision. **H. Christoph Liedtke:** Writing – review & editing, Formal analysis. **Juan E. Echevarria:** Writing – review & editing, Validation, Methodology, Data curation, Conceptualization. **Javier Juste:** Writing – review & editing, Writing – original draft, Visualization, Validation, Supervision, Project administration, Methodology, Data curation, Conceptualization.

## Declaration of competing interest

The authors declare that they have no known competing financial interests or personal relationships that could have appeared to influence the work reported in this paper.

## Data Availability

Data will be made available on request.
