# Supplementary material for: Host life history traits as drivers of viral infection in European bats
Source: Virus Res. 2026 Oct 23;373:199821. doi: 10.1016/j.virusres.2026.199821 (PMC13769332; doi:10.1016/j.virusres.2026.199821)
Supplement: Supplementary file 1 [file mmc1.docx]

**Supplementary material**

**Table S1.** Species-level traits included in the analyses and the criteria used to select representative values from the literature.

| **Traits** | **Selection criterion** |
| --- | --- |
| *Lineage diversity* | Number of genetic lineages compiled from published literature (Juste et al., 2018; Hackländer and Zachos, 2023; Froidevaux et al., 2023). |
| *Body size* | Mean forearm length (mm) calculated from individual measurements reported by Juste et al. (2018) and Froidevaux et al. (2023). |
| ***CM3*** | Mean distance from the anterior edge of the canine to the posterior edge of the third molar (mm) calculated from individual measurements reported by Juste et al. (2018) and Froidevaux et al. (2023). |
| ***Wing loading*** | Mean wing loading (g/dm^2^) calculated from individual measurements reported by Juste et al. (2018) and Froidevaux et al. (2023). |
| *Feeding behavior* | Number of prey orders constituting the primary diet of each species, compiled from Dietz and Kiefer (2017), Juste et al. (2018), Wilson and Mittermeier (2019) and Froidevaux et al. (2023). |
| *Environmental breadth* | Number of biogeographic regions occupied by each species, compiled by integrating distribution data from Juste et al. (2018), Wilson and Mittermeier (2019), Hackländer and Zachos (2023), and IUCN (2025). |
| *Group size* | Typical maximum size of summer colony, excluding isolated extreme records (Dietz and Kiefer, 2017; Juste et al., 2018; Wilson and Mittermeier, 2019; Rossetto et al., 2020; Hackländer and Zachos, 2023). |
| *Sociability* | Presence assigned when at least one publication reported mixed-species roosting (Juste et al., 2018; Wilson and Mittermeier, 2019; Rossetto et al., 2020; Hackländer and Zachos, 2023). |
| *Swarming* | Presence or absence assigned according to published evidence (Juste et al., 2018; Wilson and Mittermeier, 2019; Hackländer and Zachos, 2023). |
| ***Sampling effort*** | Total number of individual bats sampled for each species and viral group (Table S5). |

**Table S2.** Ten traits used in the analyses for each bat species. Information was compiled from Dietz and Kiefer (2017), Juste et al. (2018), Wilson and Mittermeier (2019), Rossetto et al. (2020), Hackländer and Zachos (2023), Froidevaux et al. (2023), and IUCN (2025). *Lineage diversity*: Corresponds to the number of genetic lineages identified in the phylogenetic tree constructed using nuclear or mitochondrial DNA sequences; *Body size*: Average forearm length (mm) reported for each species; ***CM3***: Linear distance from the anterior edge of the canine to the posterior edge of the third molar (mm); ***Wing loading***: Body mass divided by the total wing area (g/dm^2^); *Feeding behavior*: Number of orders that constitute the primary diet of each species; *Environmental breadth*: Number of biogeographic regions where the species occurs (Alpine, Atlantic, Black Sea, Boreal, Continental, Macaronesian, Mediterranean, Pannonic and Steppic); *Group size*: Defined as the typical upper limit individuals reported for summer roosts; *Sociability*: Tendency to share summer roosts with other bat species, value as 0 (never) or 1 (sometimes or always); Swarming: Presence (1) or absence (0) of swarming behavior; *Swarming*: Presence (1) or absence (0) of swarming behavior.

| **Species** | ***Lineage diversity*** | ***Size*** | ***CM3*** | ***Wing loading*** | ***Feeding behavior*** | ***Environmental breadth*** | ***Group size*** | ***Sociability*** | ***Swarming*** |
| --- | --- | --- | --- | --- | --- | --- | --- | --- | --- |
| *Barbastella barbastellus* | 3 | 38.74 | 4.65 | 7.87 | 5 | 8 | 100 | 0 | 1 |
| *Cnephaeus isabellinus* | 2 | 49.16 | 7.25 | 9.65 | 3 | 1 | 100 | 0 | 0 |
| *Cnephaeus nilssonii* | 2 | 39.33 | 5.45 | 10.31 | 3 | 5 | 50 | 0 | 0 |
| *Cnephaeus serotinus* | 3 | 52.59 | 7.55 | 11.91 | 3 | 8 | 60 | 0 | 0 |
| *Hypsugo savii* | 3 | 33.96 | 4.65 | 8.94 | 4 | 8 | 70 | 0 | 0 |
| *Miniopterus schreibersii* | 1 | 45.23 | 5.75 | 8.97 | 3 | 7 | 10000 | 1 | 0 |
| *Myotis alcathoe* | 1 | 31.85 | 4.9 | 6.52 | 4 | 6 | 80 | 0 | 1 |
| *Myotis bechsteinii* | 2 | 42.27 | 6.8 | 7.35 | 5 | 6 | 50 | 1 | 1 |
| *Myotis blythii* | 3 | 58.88 | 8.8 | 10.4 | 3 | 7 | 5000 | 1 | 0 |
| *Myotis brandtii* | 1 | 35.20 | 5.35 | 7.48 | 3 | 6 | 60 | 0 | 1 |
| *Myotis capaccinii* | 2 | 41.23 | 5.5 | 7.52 | 3 | 5 | 500 | 1 | 1 |
| *Myotis crypticus* | 3 | 39.31 | 6.02 | 7.38 | 3 | 5 | 50 | 0 | 1 |
| *Myotis dasycneme* | 2 | 46.39 | 6.2 | 11.48 | 3 | 5 | 100 | 1 | 1 |
| *Myotis daubentonii* | 3 | 36.92 | 5.25 | 9.11 | 7 | 8 | 50 | 1 | 1 |

**Table S2.** Continue

| **Species** | ***Lineage diversity*** | ***Size*** | ***CM3*** | ***Wing loading*** | ***Feeding behavior*** | ***Environmental breadth*** | ***Group size*** | ***Sociability*** | ***Swarming*** |
| --- | --- | --- | --- | --- | --- | --- | --- | --- | --- |
| *Myotis emarginatus* | 1 | 39.35 | 6.4 | 6.65 | 4 | 7 | 500 | 1 | 1 |
| *Myotis escalerai* | 3 | 39.31 | 5.99 | 7.38 | 4 | 2 | 1000 | 1 | 1 |
| *Myotis myotis* | 2 | 61.26 | 9.9 | 9.79 | 4 | 6 | 1000 | 1 | 0 |
| *Myotis mystacinus* | 2 | 33.90 | 5 | 7.5 | 2 | 7 | 60 | 0 | 1 |
| *Myotis nattereri* | 2 | 39.72 | 5.95 | 7.38 | 6 | 7 | 50 | 0 | 1 |
| *Myotis punicus* | 3 | 59.23 | 9.35 | 9.4 | 6 | 1 | 1000 | 0 | 0 |
| *Nyctalus lasiopterus* | 1 | 64.30 | 8.95 | 17.33 | 5 | 6 | 35 | 0 | 0 |
| *Nyctalus leisleri* | 2 | 43.49 | 5.7 | 14.15 | 6 | 9 | 50 | 0 | 0 |
| *Nyctalus noctula* | 3 | 53.99 | 7.65 | 16.8 | 3 | 8 | 50 | 0 | 0 |
| *Pipistrellus kuhlii* | 3 | 34.17 | 4.9 | 8.37 | 5 | 8 | 100 | 1 | 0 |
| *Pipistrellus nathusii* | 4 | 34.11 | 4.65 | 9.55 | 5 | 8 | 200 | 0 | 0 |
| *Pipistrellus pipistrellus* | 5 | 30.90 | 4.1 | 7.61 | 5 | 8 | 100 | 0 | 0 |
| *Pipistrellus pygmaeus* | 1 | 30.78 | 4.05 | 9.36 | 4 | 7 | 900 | 0 | 0 |
| *Plecotus auritus* | 4 | 39.29 | 5.4 | 7.13 | 5 | 8 | 50 | 1 | 1 |
| *Plecotus austriacus* | 2 | 40.20 | 4.95 | 7.23 | 6 | 8 | 30 | 1 | 1 |
| *Plecotus macrobullaris* | 4 | 41.27 | 5.6 | 7.15 | 1 | 3 | 30 | 0 | 1 |
| *Rhinolophus blasii* | 1 | 46.68 | 6.7 | 9.17 | 5 | 4 | 400 | 0 | 0 |
| *Rhinolophus euryale* | 3 | 47.83 | 6.4 | 8.51 | 4 | 6 | 1500 | 1 | 0 |
| *Rhinolophus ferrumequinum* | 5 | 55.30 | 8.7 | 10.4 | 4 | 7 | 1000 | 1 | 0 |
| *Rhinolophus hipposideros* | 8 | 37.66 | 5.25 | 5.99 | 7 | 7 | 200 | 1 | 1 |
| *Rhinolophus mehelyi* | 2 | 50.86 | 6.9 | 9.1 | 3 | 5 | 500 | 1 | 0 |
| *Tadarida teniotis* | 2 | 60.94 | 8.55 | 12.73 | 4 | 5 | 400 | 0 | 0 |
| *Vespertilio murinus* | 2 | 45.50 | 6.1 | 10.38 | 5 | 8 | 50 | 0 | 0 |

**Table S3.** ***Sampling effort*** by bat species and viral group. Values represent the total number of individuals sampled for each bat species in each viral group.

| **Species** | ***Adenoviridae*** | ***Papillomaviridae*** | ***Herpesviridae*** | ***Filoviridae*** | ***Lyssavirus***  Combined | ***Coronaviridae*** | ***Alphacoronavirus*** | ***Betacoronavirus*** | **DNA** | **RNA** | **All viruses** |
| --- | --- | --- | --- | --- | --- | --- | --- | --- | --- | --- | --- |
| *Barbastella barbastellus* | 48 | 3 | 5 | 1 | 295 | 153 | 0 | 0 | 56 | 449 | 230 |
| *Cnephaeus isabellinus* | 8 | 75 | 33 | 0 | 2203 | 13 | 0 | 8 | 116 | 2216 | 1655 |
| *Cnephaeus nilssonii* | 21 | 15 | 15 | 15 | 398 | 38 | 0 | 22 | 51 | 451 | 452 |
| *Cnephaeus serotinus* | 55 | 45 | 38 | 9 | 6607 | 171 | 3 | 0 | 139 | 6787 | 4799 |
| *Hypsugo savii* | 109 | 4 | 12 | 1 | 69 | 200 | 36 | 184 | 125 | 270 | 350 |
| *Miniopterus schreibersii* | 164 | 30 | 47 | 820 | 1409 | 330 | 161 | 0 | 241 | 2559 | 1834 |
| *Myotis alcathoe* | 4 | 0 | 1 | 0 | 47 | 23 | 0 | 0 | 5 | 70 | 74 |
| *Myotis bechsteinii* | 40 | 3 | 4 | 1 | 410 | 566 | 9 | 0 | 47 | 977 | 1020 |
| *Myotis blythii* | 39 | 5 | 6 | 3 | 160 | 64 | 32 | 0 | 50 | 227 | 166 |
| *Myotis brandtii* | 18 | 0 | 0 | 11 | 689 | 35 | 6 | 0 | 18 | 735 | 753 |
| *Myotis capaccinii* | 15 | 6 | 22 | 0 | 172 | 62 | 7 | 0 | 43 | 234 | 129 |
| *Myotis crypticus* | 0 | 0 | 0 | 0 | 0 | 2 | 2 | 0 | 0 | 2 | 2 |
| *Myotis dasycneme* | 6 | 1 | 1 | 1 | 268 | 210 | 79 | 105 | 8 | 479 | 225 |
| *Myotis daubentonii* | 336 | 219 | 243 | 217 | 7508 | 1370 | 982 | 50 | 798 | 9095 | 7463 |
| *Myotis emarginatus* | 62 | 8 | 35 | 1 | 123 | 101 | 13 | 0 | 105 | 225 | 849 |
| *Myotis escalerai* | 13 | 2 | 17 | 0 | 43 | 39 | 0 | 0 | 32 | 82 | 71 |
| *Myotis myotis* | 6261 | 6169 | 6185 | 6176 | 4342 | 6843 | 390 | 0 | 18615 | 17361 | 34608 |
| *Myotis mystacinus* | 41 | 66 | 89 | 66 | 621 | 136 | 0 | 0 | 197 | 823 | 1002 |
| *Myotis nattereri* | 107 | 57 | 62 | 55 | 2504 | 499 | 202 | 0 | 222 | 3058 | 3266 |
| *Myotis punicus* | 0 | 14 | 14 | 0 | 0 | 6 | 0 | 0 | 28 | 6 | 34 |
| *Nyctalus lasiopterus* | 183 | 0 | 3 | 0 | 0 | 185 | 174 | 0 | 186 | 185 | 371 |
| *Nyctalus leisleri* | 52 | 6 | 14 | 3 | 809 | 82 | 3 | 0 | 72 | 894 | 961 |
| *Nyctalus noctula* | 211 | 296 | 382 | 294 | 4471 | 292 | 0 | 15 | 930 | 5057 | 5532 |
| *Pipistrellus kuhlii* | 465 | 34 | 38 | 31 | 422 | 467 | 356 | 260 | 537 | 920 | 1431 |
| *Pipistrellus nathusii* | 18 | 34 | 54 | 34 | 2473 | 426 | 44 | 80 | 114 | 2933 | 3228 |
| *Pipistrellus pipistrellus* | 133 | 95 | 145 | 93 | 8498 | 1278 | 693 | 15 | 386 | 9869 | 8634 |

**Table S3.** Continue

| **Species** | ***Adenoviridae*** | ***Papillomaviridae*** | ***Herpesviridae*** | ***Filoviridae*** | ***Lyssavirus***  Combined | ***Coronaviridae*** | ***Alphacoronavirus*** | ***Betacoronavirus*** | **DNA** | **RNA** | **All viruses** |
| --- | --- | --- | --- | --- | --- | --- | --- | --- | --- | --- | --- |
| *Pipistrellus pygmaeus* | 186 | 23 | 7 | 21 | 693 | 308 | 101 | 91 | 216 | 1022 | 1223 |
| *Plecotus auritus* | 56 | 21 | 33 | 17 | 1718 | 315 | 0 | 17 | 110 | 2050 | 2000 |
| *Plecotus austriacus* | 20 | 3 | 13 | 1 | 510 | 38 | 0 | 0 | 36 | 549 | 293 |
| *Plecotus macrobullaris* | 1 | 1 | 1 | 1 | 7 | 2 | 0 | 0 | 3 | 10 | 13 |
| *Rhinolophus blasii* | 0 | 0 | 0 | 0 | 0 | 82 | 82 | 82 | 0 | 82 | 82 |
| *Rhinolophus euryale* | 52 | 2 | 52 | 0 | 44 | 288 | 243 | 246 | 106 | 332 | 164 |
| *Rhinolophus ferrumequinum* | 297 | 210 | 224 | 202 | 651 | 1448 | 110 | 264 | 731 | 2301 | 2353 |
| *Rhinolophus hipposideros* | 33 | 12 | 22 | 12 | 167 | 256 | 3 | 132 | 67 | 435 | 482 |
| *Rhinolophus mehelyi* | 1 | 4 | 3 | 0 | 0 | 35 | 15 | 13 | 8 | 35 | 33 |
| *Tadarida teniotis* | 1 | 4 | 9 | 0 | 484 | 105 | 8 | 1 | 14 | 589 | 129 |
| *Vespertilio murinus* | 225 | 209 | 210 | 209 | 262 | 26 | 0 | 0 | 644 | 497 | 1121 |

| **Species** | **Accession Numbers GenBank** |
| --- | --- |
| *Barbastella barbastellus* | JQ683176 and AF401376 |
| *Cnephaeus nilssonii* | AF376836 and AB079822 |
| *Cnephaeus serotinus* | AF376837 and DQ915023 |
| *Hypsugo savii* | AJ504450 and DQ915019 |
| *Miniopterus schreibersii* | MK737740 and MK737741 |
| *Myotis alcathoe* | JQ044687 and KF218451 |
| *Myotis bechsteinii* | AF376843 and KJ948296 |
| *Myotis blythii* | AF376840 and AY033966 |
| *Myotis brandtii* | AF376844 and AY699868 |
| *Myotis capaccinii* | AF376845 and KF218457 |
| *Myotis crypticus* | JF412410 and JX852761 |
| *Myotis dasycneme* | AF376846 and DQ915048 |
| *Myotis daubentonii* | AF376847 and AB079824 |
| *Myotis emarginatus* | AF376849 and KF218463 |
| *Myotis escalerai* | JF412390 and EU360615 |
| *Myotis myotis* | AF376860 and AF401443 |
| *Myotis mystacinus* | AF376861 and AB106583 |
| *Myotis nattereri* | AF376863 and AB106584 |
| *Myotis punicus* | AF376842 and MN158240 |
| *Nyctalus lasiopterus* | JX570900 and AF401432 |
| *Nyctalus leisleri* | JX570901 and DQ915010 |
| *Nyctalus noctula* | JX570902 and AF065103 |
| *Pipistrellus kuhlii* | AJ504444 and DQ914985 |
| *Pipistrellus nathusii* | AJ504446 and KF218497 |
| *Pipistrellus pipistrellus* | AJ504443 and DQ914967 |
| *Pipistrellus pygmaeus* | AJ504441 and AF401399 |
| *Plecotus auritus* | AB085734 and AB079817 |
| *Plecotus austriacus* | KF358491 and AF516270 |
| *Plecotus macrobullaris* | KR134409 |
| *Rhinolophus blasii* | EU436669 and KF218526 |
| *Rhinolophus euryale* | EU436671 and KF218535 |
| *Rhinolophus ferrumequinum* | AB085721 and AF143001 |
| *Rhinolophus hipposideros* | DQ297586 and DQ888658 |
| *Rhinolophus mehelyi* | EU436672 and KF218564 |
| *Tadarida teniotis* | KY581661 |
| *Vespertilio murinus* | AF376834 and AB287348 |

**Table S4. GenBank accession numbers used for phylogenetic reconstruction.** Accession numbers correspond to mitochondrial genes (cytochrome b and NADH dehydrogenase subunit 1).

(((((((((((Ppyg:0.08799954796,Ppip:0.08799954796):0.09083225274,Pkuh:0.1788318007):0.01909011169,Pnat:0.1979219124):0.01841595709,((Nlas:0.05904920372,Nnoc:0.05904920372):0.064505903,Nles:0.1235551067):0.09278276276):0.108611768,Vmur:0.3249496375):3.25059351e-07,Hsav:0.3249499626):0.08779796128,((((Paur:0.1621568558,Pmac:0.1621568558):0.0906698403,Paus:0.2528266961):0.1238887294,Bbar:0.3767154255):0.01505336952,((Cser:0.0104524711,Cnil:0.0104524711):0.1547636495,Cisa:0.1652161206):0.2265526744):0.02097912886):0.05178035577,((((((((((Mnat:0.1156611774,Mcry:0.1156611774):0.05619124264,Mesc:0.1718524201):0.001477262105,((Mpun:0.09204198289,Mbly:0.09204198289):0.002486638945,Mmyo:0.09452862184):0.07880106034):0.0582048401,(Mdau:0.1620668177,Mbec:0.1620668177):0.06946770457):0.01671123917,Mcap:0.2482457615):0.02216250463,Mmys:0.2704082661):4.096312359e-07,Mdas:0.2704086757):0.01435478711,Malc:0.2847634628):0.005028554518,Mema:0.2897920173):0.02171805232,Mbra:0.3115100697):0.15301821):0.2265468996,Msch:0.6910751792):0.01650866351,Tten:0.7075838427):0.2924161573,((((Rmeh:0.07212551074,Reur:0.07212551074):0.1857586164,Rbla:0.2578841271):0.01092578487,Rfer:0.268809912):0.08003891836,Rhip:0.3488488303):0.6511511697);

**Supplementary material 1.** Reconstructed molecular phylogeny for the 38 bat species using the cytochrome b (Cytb) and NADH dehydrogenase 1 (ND1) genes.

**Figure S1. PRISMA flow diagram of the systematic review process.** The diagram summarizes the identification, screening, eligibility, and inclusion of studies used to construct the Viral Occurrence Database and Viral Frequency Database.

**Table S5.** List of studies reporting virus-positive cases by bat species. Includes country and year of sample collection, and the diagnostic method used for virus detection. The year of sample collection may refer to studies with or without virus-negative samples. NR: not reported; ELISA: Enzyme-Linked Immunosorbent Assay; FAT: Fluorescent Antibody Test; FAVN: Fluorescent Antibody Virus Neutralization test; IHC: Immunohistochemistry; IFT: Indirect Fluorescent Antibody Test; mFAVN: Modified Fluorescent Antibody Virus Neutralization test; NGS: Next-Generation Sequencing; PCR: Polymerase Chain Reaction; RFFIT: Rabies Fluorescent Focus Inhibition Test; and RTCIT: Rapid Tissue Culture Infectivity Test.

| **Viruses** | **Host** | **Country** | **Year of sampling** | **Diagnostic test** | **Cite** |
| --- | --- | --- | --- | --- | --- |
| *Adenoviridae* | *Cnephaeus nilsonii* | Germany | 2009 | PCR | (Vidovszky et al., 2015) |
|  | *Cnephaeus serotinus* | Germany Hungary, and Italy | 2008, 2011, 2016, 2017 | PCR and NGS | (Diakoudi et al., 2019; Vidovszky et al., 2015) |
|  | *Hypsugo savii* | Italy; Spain | 2007, 2016, 2017 | PCR and NGS | (Diakoudi et al., 2019; Iglesias-Caballero et al., 2018) |
|  | *Miniopterus schreibersii* | Croatia | 2016, 2017 | NGS | (Šimić et al., 2020) |
|  | *Myotis bechsteinii* | Spain | 2007 | PCR | (Iglesias-Caballero et al., 2018) |
|  | *Myotis blythii* | Hungary | 2011 | PCR | (Vidovszky et al., 2015) |
|  | *Myotis dascycneme* | Hungary | 2011 | PCR | (Vidovszky et al., 2015) |
|  | *Myotis emarginatus* | Hungary and Spain | 2008, 2011 | PCR | (Iglesias-Caballero et al., 2018; Vidovszky et al., 2015) |
|  | *Myotis myotis* | Hungary, Spain, and Switzerland | 2004, 2007-2011, 2019-2021 | PCR and NGS | (Drexler et al., 2011; Iglesias-Caballero et al., 2018; Wiederkehr et al., 2022) |
|  | *Myotis mystacinus* | Germany and Swiss | 2008, 2015-2020 | PCR | (Hardmeier et al., 2021; Vidovszky et al., 2015) |
|  | *Myotis nattereri* | France | 2010 | NGS | (Dacheux et al., 2014) |
|  | *Nyctalus lasiopterus* | Spain | 2007 | PCR | (Iglesias-Caballero et al., 2018) |
|  | *Nyctalus leisleri* | Germany and Spain | 2007, 2008 | PCR | (Iglesias-Caballero et al., 2018; Vidovszky et al., 2015) |

**Table S5.** Continue

| **Viruses** | **Host** | **Country** | **Year of sampling** | **Diagnostic test** | **Cite** |
| --- | --- | --- | --- | --- | --- |
| *Adenoviridae* | *Nyctalus noctula* | Germany, Hungary, Spain, and Swiss | 2007, 2008, 2011, 2015-2020 | PCR and NGS | (Hardmeier et al., 2021; Iglesias-Caballero et al., 2018; Jánoska et al., 2011; Kohl et al., 2021; Vidovszky et al., 2015; Vidovszky and Boldogh, 2011) |
|  | *Pipistrellus kuhlii* | Germany, Italy, and Spain | 2009, 2016, 2017 | PCR and NGS | (Diakoudi et al., 2019; Iglesias-Caballero et al., 2018; Vidovszky et al., 2015) |
|  | *Pipistrellus nathusii* | Germany and Switzerland | 2007, 2009, 2015-2020 | PCR and NGS | (Hardmeier et al., 2021; Kohl et al., 2012; Vidovszky et al., 2015) |
|  | *Pipistrellus pipistrellus* | Germany, Italy, and Switzerland | 2007, 2008, 2009, 2015-2020 | PCR and NGS | (Diakoudi et al., 2019; Hardmeier et al., 2021; Kohl et al., 2021, 2012; Sonntag et al., 2009; Vidovszky et al., 2015) |
|  | *Pipistrellus pygmaeus* | Germany, Hungary, Spain and Switzerland | 2007, 2009, 2015-2020 | PCR and NGS | (Hardmeier et al., 2021; Iglesias-Caballero et al., 2018; Vidovszky et al., 2015) |
|  | *Plecotus auritus* | Germany | 2008 | PCR | (Vidovszky et al., 2015) |
|  | *Plecotus austriacus* | Hungary | NR | PCR | (Vidovszky and Boldogh, 2011) |
|  | *Rhinolophus euryale* | Hungary and Spain | 2004, 2007, 2008, 2011 | PCR | (Iglesias-Caballero et al., 2018; Vidovszky et al., 2015) |
|  | *Rhinolophus ferrumequinum* | Croatia, Hungary, and Spain | 2004, 2007, 2009, 2016, 2017 | PCR and NGS | (Iglesias-Caballero et al., 2018; Jánoska et al., 2011; Šimić et al., 2020; Vidovszky et al., 2015; Vidovszky and Boldogh, 2011) |
|  | *Rhinolophus hipposideros* | Hungary and Switzerland | 2010, 2019, 2021 | PCR and NGS | (Vidovszky et al., 2015; Vidovszky and Boldogh, 2011; Wiederkehr et al., 2022) |

**Table S5.** Continue

| **Viruses** | **Host** | **Country** | **Year of sampling** | **Diagnostic test** | **Cite** |
| --- | --- | --- | --- | --- | --- |
| *Adenoviridae* | *Tadarida teniotis* | Italy | 2016, 2017 | PCR and NGS | (Diakoudi et al., 2019) |
|  | *Vespertilio murinus* | Germany and Switzerland | 2007, 2019-2021 | PCR and NGS | (Vidovszky et al., 2015; Wiederkehr et al., 2022) |
| *Papillomaviridae* | *Cnephaeus isabellinus* | Spain | 2012 | PCR | (García-Pérez et al., 2014) |
|  | *Cnephaeus serotinus* | Spain and France | 2012, 2012 | PCR and NGS | (Dacheux et al., 2014; García-Pérez et al., 2014) |
|  | *Hypsugo savii* | France | 2009 | NGS | (Dacheux et al., 2014) |
|  | *Myotis brandtii* | Germany | 2020 | NGS | (Cholleti et al., 2023) |
|  | *Rhinolophus ferrumequinum* | Spain | 2008 | PCR | (García-Pérez et al., 2014) |
| *Herpesviridae* | *Cnephaeus isabellinus* | Spain | 2004, 2007 | PCR | (Pozo et al., 2016) |
|  | *Cnephaeus serotinus* | Croatia, France, Germany, Hungary, and Spain | 2002-2009, 2010, 2016, 2017 | PCR and NGS | (Dacheux et al., 2014; Molnár et al., 2008; Mühldorfer et al., 2011; Pozo et al., 2016; Šimić et al., 2020; Wibbelt et al., 2007) |
|  | *Hypsugo savii* | France and Spain | 2007, 2009 | PCR and NGS | (Dacheux et al., 2014; Pozo et al., 2016) |
|  | *Miniopterus schreibersii* | Croatia and Spain | 2002, 2004, 2007, 2016, 2017 | PCR and NGS | (Pozo et al., 2016; Šimić et al., 2020) |
|  | *Myotis alcathoe* | Spain | 2007 | PCR | (Pozo et al., 2016) |
|  | *Myotis bechsteinii* | Spain | 2007 | PCR | (Pozo et al., 2016) |
|  | *Myotis blythii* | Slovakia and Spain | 2004, NR | PCR | (Briestenská et al., 2018a; Pozo et al., 2016) |
|  | *Myotis brandtii* | Germany | 2020 | NGS | (Cholleti et al., 2023) |
|  | *Myotis capaccinii* | Spain | 2004 | PCR | (Pozo et al., 2016) |
|  | *Myotis daubentoni* | Germany and Spain | 2004, 2007, NR | PCR and NGS | (Kohl et al., 2021; Pozo et al., 2016) |

**Table S5.** Continue

| **Viruses** | **Host** | **Country** | **Year of sampling** | **Diagnostic test** | **Cite** |
| --- | --- | --- | --- | --- | --- |
| *Herpesviridae* | *Myotis emarginatus* | Croatia and Spain | 2004, 2007, 2008, 2016, 2017 | PCR and NGS | (Pozo et al., 2016; Šimić et al., 2020) |
|  | *Myotis escalerai* | Spain | 2004, 2007 | PCR | (Pozo et al., 2016) |
|  | *Myotis myotis* | Croatia, Germany, Slovakia, and Spain | 2002-2009. 2016, 2017, NR | PCR and NGS | (Briestenská et al., 2018b; Mühldorfer et al., 2011; Pozo et al., 2016; Šimić et al., 2020; Wibbelt et al., 2007) |
|  | *Myotis mystacinus* | France, Germany, and Spain | 2002-2009, NR | PCR and NGS | (Dacheux et al., 2014; Kohl et al., 2021; Mühldorfer et al., 2011; Pozo et al., 2016) |
|  | *Myotis nattereri* | France and Germany | 2002-2010 | PCR and NGS | (Dacheux et al., 2014; Mühldorfer et al., 2011; Wibbelt et al., 2007) |
|  | *Nyctalus lasiopterus* | Spain | 2007 | PCR | (Pozo et al., 2016) |
|  | *Nyctalus leisleri* | Spain | 2007 | PCR | (Pozo et al., 2016) |
|  | *Nyctalus noctula* | Germany, Spain, and Ukraine | 2002-2009, NR | PCR and NGS | (Briestenská et al., 2018b; Kohl et al., 2021; Mühldorfer et al., 2011; Pozo et al., 2016; Wibbelt et al., 2007) |
|  | *Pipistrellus kuhlii* | Spain | 2007 | PCR | (Pozo et al., 2016) |
|  | *Pipistrellus nathusii* | Germany | 2002-2009, NR | PCR | (Mühldorfer et al., 2011; Wibbelt et al., 2007) |
|  | *Pipistrellus pipistrellus* | France, Germany, and Spain | 2002-2009, NR | PCR and NGS | (Dacheux et al., 2014; Kohl et al., 2021; Mühldorfer et al., 2011; Pozo et al., 2016; Wibbelt et al., 2007) |
|  | *Pipistrellus pygmaeus* | Spain and Sweden | 2007, 2020 | PCR and NGS | (Cholleti et al., 2022; Pozo et al., 2016) |
|  | *Plecotus auritus* | Germany | 2002-2009, NR | PCR | (Mühldorfer et al., 2011; Wibbelt et al., 2007) |
|  | *Plecotus austriacus* | Spain | 2004, 2007 | PCR | (Pozo et al., 2016) |

**Table S5.** Continue

| **Viruses** | **Host** | **Country** | **Year of sampling** | **Diagnostic test** | **Cite** |
| --- | --- | --- | --- | --- | --- |
| *Herpesviridae* | *Rhinolophus ferrumequinum* | Croatia and Spain | 2004, 2007, 2008, 2016, 2017 | PCR and NGS | (Pozo et al., 2016; Šimić et al., 2020) |
|  | *Rhinolophus hipposideros* | Spain | 2007, 2008 | PCR | (Pozo et al., 2016) |
|  | *Tadarida teniotis* | Spain | 2008 | PCR | (Pozo et al., 2016) |
| *Filoviridae* | *Miniopterus schreibersii* | Hungary, Italy, and Spain | 2002, 2016-2021 | PCR, NGS, and Immunoblot | (De Arellano et al., 2019; Kemenesi et al., 2022, 2018; Negredo et al., 2011; Tóth et al., 2023) |
| *Lyssavirus* | *Barbastella barbastellus* | France and Germany | 1993-2012 | PCR and mFAVN | (Picard-Meyer et al., 2011; J. Schatz et al., 2014a) |
|  | *Cnephaeus isabellinus* | Spain | 1999-2016 | PCR, NGS, and RFFIT | (Marston et al., 2015; Mingo-Casas et al., 2018; Vázquez-Morón et al., 2011, 2008b, 2008a) |

**Table S5.** Continue

| **Viruses** | **Host** | **Country** | **Year of sampling** | **Diagnostic test** | **Cite** |
| --- | --- | --- | --- | --- | --- |
| *Lyssavirus* | *Cnephaeus serotinus* | Croatia, Denmark, England, France, Germany, Luxembourg, Netherlands, Poland, Russia, Slovakia, Slovenia Spain, and Ukraine | 1968, 1970, 1979, 1982, 1985-2020 | PCR, NGS, FAT, RTCIT, FAVN, IFT, IHC, mFAVN, and RFFIT | (Amengual et al., 1997; Badrane et al., 2001; Begeman et al., 2020; Bourhy et al., 2005, 1992; Calvelage et al., 2021; Dacheux et al., 2009; Davis et al., 2005; Delbos et al., 2006; Delmas et al., 2008; Folly et al., 2021; Freuling et al., 2012; Harris et al., 2009; Klein et al., 2021; López-Roig et al., 2014; Marston et al., 2013, 2007; Mingo-Casas et al., 2018; Mühldorfer et al., 2011; Müller et al., 2007; Nieuwenhuijs et al., 1992; Ondrejková et al., 2004; Parize et al., 2020; Pérez-Jordá et al., 1995; Picard-Meyer et al., 2017, 2014, 2011, 2004; Robardet et al., 2017; J. Schatz et al., 2014a, 2014b; Juliane Schatz et al., 2014; Serra-Cobo et al., 2013; Servat et al., 2015; Šimić et al., 2018; Smreczak et al., 2009; Takumi et al., 2009; Troupin et al., 2017; Van Der Poel et al., 2005; Van Gucht et al., 2013; Sonia Vázquez-Morón et al., 2006; S. Vázquez-Morón et al., 2006) |

**Table S5.** Continue

| **Viruses** | **Host** | **Country** | **Year of sampling** | **Diagnostic test** | **Cite** |
| --- | --- | --- | --- | --- | --- |
| *Lyssavirus* | *Hypsugo savii* | Spain | 2001-2012 | RFFIT | (López-Roig et al., 2014; Serra-Cobo et al., 2013) |
|  | *Miniopterus schreibersii* | Croatia, France, Russia, and Spain | 1996, 1998-2010, 2016, 2017 | PCR, NGS, RFFIT, ELISA, FAT, RTCIT, and mFAVN | (Botvinkin et al., n.d.; Ceballos et al., 2013; Kuzmin et al., 2005; Picard-Meyer et al., 2019, 2011; Serra-Cobo et al., 2013, 2002; Šimić et al., 2018) |
|  | *Myotis blythii* | Croatia, France, and Spain | 2001-2010, 2016, 2017 | mFAVN and RFFIT | (Picard-Meyer et al., 2011; Serra-Cobo et al., 2013; Šimić et al., 2018) |
|  | *Myotis brandtii* | Finland | 2017 | PCR and RTCIT | (Nokireki et al., 2018) |
|  | *Myotis capaccinii* | Slovenia and Spain | 2001-2010, 2012-2019 | PCR, NGS, FAT, RTCIT, and RFFIT | (Černe et al., 2023; Serra-Cobo et al., 2013) |
|  | *Myotis dasycneme* | England, Netherlands | 1986-1991, 1993-2003, ND | PCR, NGS, FAT, and IFT | (Amengual et al., 1997; Badrane et al., 2001; Bourhy et al., 2005; Davis et al., 2005; Delmas et al., 2008; Nieuwenhuijs et al., 1992; Van Der Poel et al., 2005) |

**Table S5.** Continue

| **Viruses** | **Host** | **Country** | **Year of sampling** | **Diagnostic test** | **Cite** |
| --- | --- | --- | --- | --- | --- |
| *Lyssavirus* | *Myotis daubentoni* | England, Finland, Germany, Norway, Scotland, Spain, Sweden, Switzerland, and United Kingdom | 1976-2013, 2015, 2016 | PCR, FAT, RTCIT, FAVN, IHC, mFAVN, and RFFIT | (Amengual et al., 1997; Banyard et al., 2009; Bourhy et al., 2005; Davis et al., 2005; Fooks et al., 2006, 2003; Freuling et al., 2008; Harris et al., 2009, 2007; Horton et al., 2020; Jakava-Viljanen et al., 2010; Johnson et al., 2006, 2003; Kappeier Andreas, 1992; Miia et al., 2015; Moldal et al., 2017; Nokireki et al., 2017, 2013; J. Schatz et al., 2014b; Juliane Schatz et al., 2014; Serra-Cobo et al., 2013) |
|  | *Myotis escalerai* | Spain | 2001-2010 | RFFIT | (Serra-Cobo et al., 2013) |
|  | *Myotis myotis* | Croatia, France, Germany, and Spain | 1995-2012, 2016, 2017 | PCR, FAT, mFAVN, and RFFIT | (Amengual et al., 2007; Picard-Meyer et al., 2011; J. Schatz et al., 2014a; Serra-Cobo et al., 2013, 2002; Šimić et al., 2018) |
|  | *Myotis nattereri* | Croatia, France, Germany, Poland, and Spain | 1993-2013, 2016, 2020 | PCR, NGS, FAT, RTCIT, and mFAVN | (Freuling et al., 2013, 2011; Klein et al., 2021; Picard-Meyer et al., 2014, 2013; J. Schatz et al., 2014a; Serra-Cobo et al., 2002; Šimić et al., 2018; Smreczak et al., 2020, 2018) |
|  | *Nyctalus noctula* | Serbia | ND | Neutralization test | (Nikolić and Jelesić, 1956) |

**Table S5.** Continue

| **Viruses** | **Host** | **Country** | **Year of sampling** | **Diagnostic test** | **Cite** |
| --- | --- | --- | --- | --- | --- |
| *Lyssavirus* | *Pipistrellus kuhlii* | France and Spain | 2001-2016 | FAT, RTCIT, and RFFIT | (López-Roig et al., 2014; Parize et al., 2020; Serra-Cobo et al., 2013) |
|  | *Pipistrellus nathusii* | Germany | 1992, 1998-2013 | PCR, FAT, IHC, and RTCIT | (Freuling et al., 2012; J. Schatz et al., 2014b; Juliane Schatz et al., 2014) |
|  | *Pipistrellus pipistrellus* | France, Germany, Slovakia, and Spain | 1994, 1998-202 | PCR, FAT, RTCIT, and RFFIT | (Freuling et al., 2012; Klein et al., 2021; Korytár et al., 2022; López-Roig et al., 2014; Müller et al., 2007; Parize et al., 2020; Juliane Schatz et al., 2014; Serra-Cobo et al., 2013) |
|  | *Pipistrellus pygmaeus* | Denmark and Sweden | 1998, 2020 | NGS | (Calvelage et al., 2021; Cholleti et al., 2022) |
|  | *Plecotus auritus* | Germany | 1996, 1998-2013 | PCR, FAT, and RTCIT | (Freuling et al., 2012; Müller et al., 2007; Juliane Schatz et al., 2014) |
|  | *Plecotus austriacus* | Spain | 2001-2012 | RFFIT | (Serra-Cobo et al. 2013; López-Roig et al. 2014) |
|  | *Rhinolophus ferrumequinum* | Croatia, France, and Spain | 1997, 2000-2010, 2016, 2017 | PCR, Mfavn, and RFFIT | (Serra-Cobo et al. 2002; Evelyne Picard-Meyer et al. 2011; Serra-Cobo et al. 2013; Šimić et al. 2018) |
|  | *Tadarida teniotis* | Spain | 1997, 2001-2010, 2012 | PCR and RFFIT | (Serra-Cobo et al. 2002, 2013; López-Roig et al. 2014) |
|  | *Vespertilio murinus* | Ukraine | 1987, 2012, 2013 | PCR | (B Amengual et al. 1997; Davis et al. 2005) |
| *Coronaviridae* | *Cnephaeus isabellinus* | Spain | 2004-2007, 2020 | PCR | (Falcón et al. 2011; Moraga-Fernández et al. 2022) |
|  | *Cnephaeus nilssonii* | Finland | 2014-2016 | PCR | (Kivistö et al. 2020) |
|  | *Cnephaeus serotinus* | Denmark, France, Italy, Russia, and Spain | 2008-2017, 2020, 2021 | PCR and NGS | (Ar Gouilh et al. 2018; Lazov et al. 2018; Moraga-Fernández et al. 2022; Popov et al. 2023; De Benedictis et al. 2014) |

**Table S5.** Continue

| **Viruses** | **Host** | **Country** | **Year of sampling** | **Diagnostic test** | **Cite** |
| --- | --- | --- | --- | --- | --- |
| *Coronaviridae* | *Hypsugo savii* | Italy and Spain | 2004-2012, 2020-2022 | PCR and NGS | (Falcón et al., 2011; Lelli et al., 2013; Leopardi et al., 2023; Moraga-Fernández et al., 2022; Moreno et al., 2017) |
|  | *Miniopterus schreibersii* | Bulgaria, France, Portugal, and Spain | 2004-2016, 2020, 2022 | PCR | (Drexler et al., 2010; Falcón et al., 2011; Gouilh et al., 2018; Hemnani et al., 2023; Monchatre-Leroy et al., 2017; Moraga-Fernández et al., 2022) |
|  | *Myotis bechsteinii* | Germany and Spain | 2007, 2020 | PCR | (Gloza-Rausch et al., 2008; Moraga-Fernández et al., 2022) |
|  | *Myotis blythii* | Italy and Spain | 2004-2007, 2009-2016, 2020 | PCR | (De Benedictis et al., 2014; Falcón et al., 2011; Moraga-Fernández et al., 2022; Rizzo et al., 2017) |
|  | *Myotis brandtii* | Finland and Russia | 2014-2016 | PCR and NGS | (Kivistö et al., 2020; Speranskaya et al., 2023) |
|  | *Myotis capaccinii* | Spain | 2008-2016, 2020 | PCR | (Gouilh et al., 2018; Moraga-Fernández et al., 2022) |
|  | *Myotis crypticus* | Italy | 2020-2022 | PCR | (Leopardi et al., 2023) |
|  | *Myotis dasycneme* | Denmark, Germany, Netherlands, and Russia | 2007, 2009, 2013-2017 | PCR and NGS | (Gloza-Rausch et al., 2008; Lazov et al., 2021, 2018; Reusken et al., 2010; Speranskaya et al., 2023) |

**Table S5.** Continue

| **Viruses** | **Host** | **Country** | **Year of sampling** | **Diagnostic test** | **Cite** |
| --- | --- | --- | --- | --- | --- |
| *Coronaviridae* | *Myotis daubentoni* | Denmark, Finland, France, Germany, Hungary, Italy, Netherlands, Poland, Russia, Spain, Sweden, and United Kingdom | 2004-2018, 2020-2022 | PCR and NGS | (Arnaout et al., 2023; August et al., 2012; Falcón et al., 2011; Gloza-Rausch et al., 2008; Gouilh et al., 2018; Kemenesi et al., 2014; Kivistö et al., 2020; Lazov et al., 2021, 2018; Lwande et al., 2022; Moraga-Fernández et al., 2022; Orłowska et al., 2022; Reusken et al., 2010; Rizzo et al., 2017; Speranskaya et al., 2023) |
|  | *Myotis emarginatus* | France and Spain | 2014, 2017, 2018, 2020 | PCR | (Arnaout et al., 2023; Monchatre-Leroy et al., 2017; Moraga-Fernández et al., 2022) |
|  | *Myotis escalerai* | Spain | 2020 | PCR | (Moraga-Fernández et al., 2022) |
|  | *Myotis myotis* | Germany, Spain, France, Hungary, Italy, Portugal, and Switzerland | 2004-2022 | PCR and NGS | (Arnaout et al., 2023; Drexler et al., 2011; Falcón et al., 2011; Gouilh et al., 2018; Hemnani et al., 2023; Kemenesi et al., 2014; Moraga-Fernández et al., 2022; Rizzo et al., 2017; Wiederkehr et al., 2022) |
|  | *Myotis mystacinus* | Spain | 2020 | PCR | (Moraga-Fernández et al., 2022) |
|  | *Myotis nattereri* | Germany, Denmark, France, Hungary, Italy, and United Kingdom | 2006-2017 | PCR | (August et al., 2012; Fischer et al., 2016; Gouilh et al., 2018; Kemenesi et al., 2014; Lazov et al., 2018; Monchatre-Leroy et al., 2017; Rizzo et al., 2017) |
|  | *Nyctalus lasiopterus* | Spain | 2004-2007, 2020 | PCR | (Fischer et al., 2016; Moraga-Fernández et al., 2022) |
|  | *Nyctalus leisleri* | Bulgaria and Spain | 2008, 2020 | PCR | (Drexler et al., 2010; Moraga-Fernández et al., 2022) |

**Table S5.** Continue

| **Viruses** | **Host** | **Country** | **Year of sampling** | **Diagnostic test** | **Cite** |
| --- | --- | --- | --- | --- | --- |
| *Coronaviridae* | *Nyctalus noctula* | Italy, Netherlands, Russia, and Switzerland | 2009, 2010-2012, 2015-2020 | PCR and NGS | (Hardmeier et al., 2021; Lelli et al., 2013; Reusken et al., 2010; Speranskaya et al., 2023) |
|  | *Pipistrellus kuhlii* | Spain, Italy, and Russia | 2004-2007, 2010-2016, 2020-2022 | PCR and NGS | (De Sabato et al., 2019; Falcón et al., 2011; Lelli et al., 2013; Leopardi et al., 2023; Moreno et al., 2017; Popov et al., 2023; Rizzo et al., 2017) |
|  | *Pipistrellus nathusii* | Germany, Netherlands, Romania, Russia, and Ukraine | 2007, 2009-2013, 2015, 2017-2019 | PCR and NGS | (Annan et al., 2013; Fischer et al., 2016; Gloza-Rausch et al., 2008; Ortiz et al., 2023; Speranskaya et al., 2023) |
|  | *Pipistrellus pipistrellus* | Spain, France, Italy, Netherlands, and Romania | 2009, 2014, 2015-2022 | PCR | (Annan et al., 2013; Goffard et al., 2015; Leopardi et al., 2023; Monchatre-Leroy et al., 2017; Moraga-Fernández et al., 2022; Ortiz et al., 2023; Reusken et al., 2010; Rizzo et al., 2017) |
|  | *Pipistrellus pygmaeus* | Germany, Denmark, Hungary, Romania and Sweden | 2007, 2009-2017, 2020, ND | PCR and NGS | (Annan et al., 2013; Cholleti et al., 2022; Fischer et al., 2016; Gloza-Rausch et al., 2008; Kemenesi et al., 2014; Kohl et al., 2021; Lazov et al., 2021, 2018) |
|  | *Plecotus auritus* | Spain and Italy | 2013-2016, 2020-2022 | PCR | (Lecis et al., 2019; Leopardi et al., 2023; Moraga-Fernández et al., 2022; Rizzo et al., 2017) |
|  | *Rhinolophus blasii* | Bulgaria | 2008 | PCR | (Drexler et al., 2010; Sander et al., 2022) |
|  | *Rhinolophus euryale* | Bulgaria, Spain, and Hungary | 2008, 2009, 2012, 2013, 2020 | PCR | (Drexler et al., 2010; Kemenesi et al., 2014; Moraga-Fernández et al., 2022; Sander et al., 2022) |

**Table S5.** Continue

| **Viruses** | **Host** | **Country** | **Year of sampling** | **Diagnostic test** | **Cite** |
| --- | --- | --- | --- | --- | --- |
| *Coronaviridae* | *Rhinolophus ferrumequinum* | Bulgaria, Spain, France, Hungary, Italy, Portugal, and Russia | 2008-2018, 2020, 2022 | PCR and NGS | (Alkhovsky et al., 2022; Arnaout et al., 2023; Balboni et al., 2012, 2011; Drexler et al., 2010; Gouilh et al., 2018; Hemnani et al., 2023; Kemenesi et al., 2014; Lecis et al., 2019; Moraga-Fernández et al., 2022; Rizzo et al., 2017; Sander et al., 2022) |
|  | *Rhinolophus hipposideros* | Slovenia, Spain, Hungary, Italy, Poland, and Russia | 2008-2013, 2020-2022 | PCR and NGS | (Alkhovsky et al., 2022; Kemenesi et al., 2014; Lelli et al., 2013; Orłowska et al., 2022; Rihtarič et al., 2010; Sander et al., 2022) |
|  | *Rhinolophus mehelyi* | Bulgaria and Portugal | 2008, 2022 | PCR | (Drexler et al., 2010; Hemnani et al., 2023) |
|  | *Tadarida teniotis* | Spain and Italy | 2008-2016 | PCR | (Gouilh et al., 2018; Lecis et al., 2019) |
|  | *Vespertilio murinus* | Switzerland | 2015-2021 | PCR and NSG | (Hardmeier et al., 2021; Wiederkehr et al., 2022) |

**Table S6.** Number of individuals sampled (n) and number of positive detections (+) for each bat species and viral group used to estimate viral frequency (Fig. 2). NE indicates species–virus combinations that were not studied or for which data were not reported.

| **Species** | ***Adenoviridae*** | | ***Papillomaviridae*** | | ***Herpesviridae*** | | ***Filoviridae*** | | ***Lyssavirus***  Combined | | ***Coronaviridae*** | | ***Alphacoronavirus*** | | ***Betacoronavirus*** | | **DNA** | | **RNA** | | **All viruses** | |
| --- | --- | --- | --- | --- | --- | --- | --- | --- | --- | --- | --- | --- | --- | --- | --- | --- | --- | --- | --- | --- | --- | --- |
|  | + | n | + | n | + | n | + | n | + | n | + | n | + | n | + | n | + | n | + | n | + | n |
| *Barbastella barbastellus* | 0 | 48 | 0 | 3 | 0 | 5 | 0 | 1 | 2 | 295 | 0 | 153 | NE | NE | NE | NE | 0 | 56 | 2 | 449 | 2 | 505 |
| *Cnephaeus isabellinus* | 0 | 8 | 7 | 75 | 16 | 33 | NE | NE | 111 | 2203 | 2 | 13 | NE | NE | 1 | 8 | 23 | 116 | 113 | 2216 | 136 | 2332 |
| *Cnephaeus nilssonii* | 1 | 21 | 0 | 15 | 0 | 15 | 0 | 15 | 0 | 398 | 2 | 38 | NE | NE | 1 | 22 | 1 | 51 | 2 | 451 | 3 | 502 |
| *Cnephaeus serotinus* | 17 | 55 | 18 | 45 | 15 | 39 | 0 | 9 | 1379 | 6607 | 8 | 171 | 3 | 3 | NE | NE | 50 | 139 | 1387 | 6787 | 1437 | 6926 |
| *Hypsugo savii* | 11 | 109 | 1 | 4 | 3 | 12 | 0 | 1 | 10 | 69 | 12 | 200 | 1 | 36 | 8 | 184 | 15 | 125 | 22 | 270 | 37 | 395 |
| *Miniopterus schreibersii* | 0 | 164 | 0 | 30 | 29 | 47 | 37 | 820 | 282 | 1409 | 62 | 330 | 48 | 161 | NE | NE | 29 | 241 | 381 | 2559 | 410 | 2800 |
| *Myotis alcathoe* | 0 | 4 | NE | NE | 1 | 1 | NE | NE | 0 | 47 | 0 | 23 | NE | NE | NE | NE | 1 | 5 | 0 | 70 | 1 | 75 |
| *Myotis bechsteinii* | 1 | 40 | 0 | 3 | 2 | 4 | 0 | 1 | 0 | 410 | 11 | 566 | 1 | 9 | NE | NE | 3 | 47 | 11 | 977 | 14 | 1024 |
| *Myotis blythii* | 1 | 39 | 0 | 5 | 6 | 6 | 0 | 3 | 31 | 160 | 7 | 64 | 2 | 32 | NE | NE | 7 | 50 | 38 | 227 | 45 | 277 |
| *Myotis brandtii* | 0 | 18 | NE | NE | NE | NE | 0 | 11 | 2 | 689 | 2 | 35 | 2 | 6 | NE | NE | 0 | 18 | 4 | 735 | 4 | 753 |
| *Myotis capaccinii* | 0 | 15 | 0 | 6 | 3 | 22 | NE | NE | 21 | 172 | 19 | 62 | 7 | 7 | NE | NE | 3 | 43 | 40 | 234 | 43 | 277 |
| *Myotis crypticus* | NE | NE | NE | NE | NE | NE | NE | NE | NE | NE | 1 | 2 | 1 | 2 | NE | NE | NE | NE | 1 | 2 | 1 | 2 |
| *Myotis dasycneme* | 2 | 6 | 0 | 1 | 0 | 1 | 0 | 1 | 26 | 268 | 43 | 210 | 17 | 79 | 20 | 105 | 2 | 8 | 69 | 479 | 71 | 487 |
| *Myotis daubentonii* | 0 | 336 | 0 | 219 | 12 | 243 | 0 | 217 | 234 | 7508 | 149 | 1370 | 132 | 982 | 8 | 50 | 12 | 798 | 383 | 9095 | 395 | 9893 |
| *Myotis emarginatus* | 4 | 62 | 0 | 8 | 7 | 35 | 0 | 1 | 0 | 123 | 10 | 101 | 4 | 13 | NE | NE | 11 | 105 | 10 | 225 | 21 | 330 |
| *Myotis escalerai* | 0 | 13 | 0 | 2 | 9 | 17 | NE | NE | 6 | 43 | 4 | 39 | NE | NE | NE | NE | 9 | 32 | 10 | 82 | 19 | 114 |
| *Myotis myotis* | 5 | 6261 | 0 | 6169 | 11 | 6185 | 0 | 6176 | 652 | 4342 | 84 | 6843 | 62 | 390 | NE | NE | 16 | 18615 | 736 | 17361 | 752 | 35976 |
| *Myotis mystacinus* | 1 | 41 | 0 | 66 | 4 | 90 | 0 | 66 | 0 | 621 | 1 | 136 | NE | NNE | NE | NE | 5 | 197 | 1 | 823 | 6 | 1020 |
| *Myotis nattereri* | 1 | 107 | 0 | 57 | 2 | 58 | 0 | 55 | 10 | 2504 | 36 | 499 | 36 | 202 | NE | NE | 3 | 222 | 46 | 3058 | 49 | 3280 |
| *Myotis punicus* | NE | NE | 0 | 14 | 0 | 14 | NE | NE | NE | NE | 0 | 6 | NE | NE | NE | NE | 0 | 28 | 0 | 6 | 0 | 34 |
| *Nyctalus lasiopterus* | 16 | 183 | NE | NE | 3 | 3 | NE | NE | NE | NE | 15 | 185 | 5 | 174 | NE | NE | 19 | 186 | 15 | 185 | 19 | 371 |
| *Nyctalus leisleri* | 5 | 52 | 0 | 6 | 6 | 14 | 0 | 3 | 0 | 809 | 9 | 82 | 1 | 3 | NE | NE | 11 | 72 | 9 | 894 | 20 | 966 |
| *Nyctalus noctula* | 33 | 211 | 0 | 296 | 18 | 423 | 0 | 294 | 3 | 4471 | 6 | 292 | NE | NE | 6 | 15 | 51 | 930 | 9 | 5057 | 60 | 5987 |
| *Pipistrellus kuhlii* | 24 | 465 | 0 | 34 | 5 | 38 | 0 | 31 | 5 | 422 | 23 | 467 | 11 | 356 | 6 | 260 | 29 | 537 | 28 | 920 | 57 | 1457 |

**Table S6.** Continue

| **Species** | ***Adenoviridae*** | | ***Papillomaviridae*** | | ***Herpesviridae*** | | ***Filoviridae*** | | ***Lyssavirus***  Combined | | ***Coronaviridae*** | | ***Alphacoronavirus*** | | ***Betacoronavirus*** | | **DNA** | | **RNA** | | **All viruses** | |
| --- | --- | --- | --- | --- | --- | --- | --- | --- | --- | --- | --- | --- | --- | --- | --- | --- | --- | --- | --- | --- | --- | --- |
|  | + | n | + | n | + | n | + | n | + | n | + | n | + | n | + | n | + | n | + | n | + | n |
| *Pipistrellus nathusii* | 2 | 18 | 0 | 34 | 9 | 62 | 0 | 34 | 6 | 2473 | 80 | 426 | 4 | 44 | 30 | 80 | 11 | 114 | 86 | 2933 | 97 | 3047 |
| *Pipistrellus pipistrellus* | 11 | 133 | 0 | 95 | 40 | 158 | 2 | 93 | 20 | 8498 | 168 | 1278 | 157 | 693 | 3 | 15 | 51 | 386 | 190 | 9869 | 241 | 10255 |
| *Pipistrellus pygmaeus* | 19 | 186 | 0 | 23 | 1 | 7 | 0 | 21 | 1 | 693 | 24 | 308 | 16 | 101 | 9 | 91 | 20 | 216 | 25 | 1022 | 45 | 1238 |
| *Plecotus auritus* | 1 | 56 | 0 | 21 | 2 | 33 | 0 | 17 | 5 | 1718 | 6 | 315 | NE | NE | 3 | 17 | 3 | 110 | 11 | 2050 | 14 | 2160 |
| *Plecotus austriacus* | 1 | 20 | 0 | 3 | 9 | 13 | 0 | 1 | 39 | 510 | 0 | 38 | NE | NE | NE | NE | 10 | 36 | 39 | 549 | 49 | 585 |
| *Plecotus macrobullaris* | 0 | 1 | 0 | 1 | 0 | 1 | 0 | 1 | 0 | 7 | 0 | 2 | NE | NE | NE | NE | 0 | 3 | 0 | 10 | 0 | 13 |
| *Rhinolophus blasii* | NE | NE | NE | NE | NE | NE | NE | NE | NE | NE | 23 | 82 | 7 | 82 | 16 | 82 | NE | NE | 23 | 82 | 23 | 82 |
| *Rhinolophus euryale* | 7 | 52 | 0 | 2 | 0 | 52 | NE | NE | 0 | 44 | 124 | 288 | 25 | 243 | 87 | 246 | 7 | 106 | 124 | 332 | 131 | 438 |
| *Rhinolophus ferrumequinum* | 13 | 297 | 1 | 210 | 15 | 224 | 0 | 202 | 121 | 651 | 235 | 1448 | 18 | 110 | 55 | 264 | 29 | 731 | 356 | 2301 | 385 | 3032 |
| *Rhinolophus hipposideros* | 3 | 33 | 0 | 12 | 1 | 22 | 0 | 12 | 0 | 167 | 41 | 256 | 1 | 3 | 38 | 132 | 4 | 67 | 41 | 435 | 45 | 502 |
| *Rhinolophus mehelyi* | 0 | 1 | 0 | 4 | 0 | 3 | NE | NE | NE | NE | 8 | 35 | 4 | 15 | 4 | 13 | 0 | 8 | 8 | 35 | 8 | 43 |
| *Tadarida teniotis* | 1 | 1 | 0 | 4 | 6 | 9 | NE | NE | 74 | 484 | 2 | 105 | 1 | 8 | 1 | 1 | 7 | 14 | 76 | 589 | 83 | 603 |
| *Vespertilio murinus* | 1 | 225 | 0 | 209 | 0 | 210 | 0 | 209 | 3 | 262 | 0 | 26 | NE | NE | NE | NE | 1 | 644 | 3 | 497 | 4 | 1141 |

**Table S7.** Phylogenetic signal (λ) estimates and associated p-values for host traits across different viral groupings. Coefficients (β), along with their standard errors (SE), 95% confidence intervals (CI lower and CI upper), t-statistics (t), p-values, and degrees of freedom (df). When the Shapiro–Wilk normality test indicated non-normality of log₁₀-transformed data, additional t-statistic and p-values obtained after Box–Cox transformation are reported.

|  | ***Adenoviridae*** | ***Papillomaviridae*** | ***Herpesviridae*** | ***Lyssavirus***  Active infection | ***Lyssavirus***  Serological | ***Lyssavirus***  Combined |
| --- | --- | --- | --- | --- | --- | --- |
| **λ** | λ= 0.8912  p-value 0.0011 | λ= 7.4088e-05  p-value 1 | λ= 7.4088e-05  p-value 1 | λ= 7.4088e-05  p-value 1 | λ= 0.9824  p-value 0.4743 | λ= 7.4088e-05  p-value 1 |
| **Log10** | | | | | | |
| *Lineage diversity* | β 0.5437  SE 2.8090  CI lower -4.9618  CI upper 6.0493  t -0.1936  p-value 0.8480 | β 5.3503  SE 3.4995  CI lower -1.5087  CI upper 12.2093  t 1.5289  p-value 0.1394 | β 4.1887  SE 3.3083  CI lower -2.2946  CI upper 10.6730  t 1.2661  p-value 0.2166 | β 4.3102  SE 5.1029  CI lower -5.6915  CI upper 14.3118  t 0.8447  p-value 0.04070 | β 11.7179  SE 4.3575  CI lower 3.1772  CI upper 20.2585  t 2.6891  p-value 0.0141 | β 7.5003  SE 3.7688  CI lower 3.1134  CI upper 14.8873  t 1.9901  p-value 0.0480 |
| *Body size* | β -1.9615  SE 7.1293  CI lower -15.9349  CI upper 12.0119  t -0.2751  p-value 0.7854 | β 42.1354  SE 8.4298  CI lower 25.6129  CI upper 58.6579  t 4.9984  p-value 4.17e-05 | β 33.4393  SE 8.5204  CI lower 16.7393  CI upper 50.1393  t 3.9246  p-value 0.0005 | β 46.2856  SE 11.8766  CI lower 23.0075  CI upper 69.5637  t 3.8972  p-value 0.0003 | β 33.6729  SE 12.4355  CI lower 9.2993  CI upper 58.0465  t 2.7078  p-value 0.0135 | β 42.9924  SE 9.4251  CI lower 24.5192  CI upper 61.4656  t 4.5615  p-value 0.0001 |
| *Feeding behavior* | β 4.0259  SE 4.6296  CI lower -5.0481  CI upper 13.0999  t 0.8696  p-value 0.3925 | β 2.1721  SE 4.1870  CI lower -6.0344  CI upper 10.3785  t 0.5188  p-value 0.6087 | β 5.0243  SE 4.7791  CI lower -4.3427  CI upper 14.3913  t 1.0513  p-value 0.3027 | β 12.8703  SE 7.1545  CI lower -1.1526  CI upper 26.8932  t 1.7989  p-value 0.0852 | β 0.6507  SE 8.7688  CI lower -16.5362  CI upper 17.8362  t 0.0742  p-value 0.9416 | β 9.6664  SE 5.5449  CI lower -1.2016  CI upper 20.5344  t 1.7433  p-value 0.0940 |
| *Environmental breadth* | β 8.5131  SE 3.4951  CI lower 1.6627  CI upper 15.3636  t 2.4357  p-value 0.0220 | β 3.6384  SE 2.7331  CI lower -1.7185  CI upper 8.9953  t 1.3312  p-value 0.1956 | β 9.6008  SE 3.1410  CI lower 3.4445  CI upper 15.7571  t 3.0566  p-value 0.0051 | β 1.3436  SE 5.8369  CI lower -10.0968  CI upper 12.7840  t 0.2302  p-value 0.8200 | β 5.5377  SE 7.2599  CI lower -8.6917  CI upper 19.7671  t 0.7628  p-value 0.4545 | β 2.2245  SE 3.9910  CI lower -5.5978  CI upper 10.0468  t 0.5574  p-value 0.5824 |
| *Group size* | β 0.1658  SE 1.5972  CI lower -2.9647  CI upper 3.2962  t 0.1038  p-value 0.9181 | β 1.7410  SE 1.6830  CI lower -1.5578  CI upper 5.0397  t 1.0344  p-value 0.3112 | β 3.2762  SE 1.9476  CI lower -0.5411  CI upper 7.0936  t 1.6822  p-value 0.1045 | β 2.4762  SE 2.6310  CI lower -2.6806  CI upper 7.6329  t 0.9412  p-value 0.3564 | β 1.3128  SE 2.1677  CI lower -2.9358  CI upper 5.5615  t 0.6056  p-value 0.5516 | β 3.8769  SE 1.9029  CI lower -0.1471  CI upper 7.6066  t 2.0373  p-value 0.0527 |
| *Sociability* | β 0.3511  SE 2.0004  CI lower -3.5696  CI upper 4.2719  t 0.1755  p-value 0.8620 | β -2.2745  SE 1.9266  CI lower -6.0505  CI upper 1.5015  t -1.1806  p-value 0.2493 | β -0.7433  SE 2.1881  CI lower -5.0319  CI upper 3.5454  t -0.3397  p-value 0.7368 | β -3.0081  SE 2.8329  CI lower -8.5606  CI upper 2.5444  t -1.0619  p-value 0.2993 | β 0.7311  SE 2.6915  CI lower -4.5441  CI upper 6.0064  t 0.2717  p-value 0.7887 | β -1.9534  SE 2.2470  CI lower -6.3576  CI upper 2.4507  t -0.8693  p-value 0.3932 |
| *Swarming* | β -2.6487  SE 2.5581  CI lower -7.6626  CI upper 2.3652  t -1.0354  p-value 0.3100 | β 3.9372  SE 2.7093  CI lower -1.3730  CI upper 9.2475  t 1.4532  p-value 0.1591 | β 6.2991  SE 3.1642  CI lower 0.0972  CI upper 12.5011  t 1.9907  p-value 0.0571 | β 5.4694  SE 3.8339  CI lower -2.0450  CI upper 12.9838  t 1.4266  p-value 0.1671 | β 3.2120  SE 3.1815  CI lower -3.0237  CI upper 9.4476  t 1.0096  p-value 0.3248 | β 4.5834  SE 2.8178  CI lower -0.9395  CI upper 10.1063  t 1.6266  p-value 0.1168 |
| **df** | 26 | 24 | 26 | 23 | 20 | 24 |
| **Shapiro-Wilk normality test** | W= 0.91819  p-value 0.0592 | W= 0.9378  p-value 0.0650 | W= 0.9872  p-value 0.9543 | W= 0.9773  p-value 0.7402 | W= 0.9638  p-value 0.4267 | W= 0.9722  p-value 0.5647 |
| **BOXCOX** | | | | | | |
| *Lineage diversity* | / | / | / | / | / | / |
| *Body size* | / | / | / | / | / | / |
| *Feeding behavior* | / | / | / | / | / | / |
| *Environmental breadth* | / | / | / | / | / | / |
| *Group size* | / | / | / | / | / | / |
| *Sociability* | / | / | / | / | / | / |
| *Swarming* | / | / | / | / | / | / |
| **Shapiro-Wilk normality test** | / | / | / | / | / | / |

**Table S7.** Continue

|  | ***Coronaviridae*** | ***Alphacoronavirus*** | ***Betacoronavirus*** | **DNA** | **RNA** | **All viruses** | |
| --- | --- | --- | --- | --- | --- | --- | --- |
| **λ** | λ= 0.3015  p-value 0.0982 | λ= 7.9012e-05  p-value 1 | λ= 1.0622  p-value 0.0114 | λ= 7.4088e-05  p-value 1 | λ= 0.2947  p-value 0.0647 | λ= 7.4088e-05  p-value 1 | |
| **Log10** | | | | | | |  |
| *Lineage diversity* | β -0.8696  SE 1.9108  CI lower -4.6148  CI upper 2.8756  t -0.4551  p-value 0.6524 | β 0.1476  SE 0.4235  CI lower -0.6824  CI upper 0.9776  t 0.3486  p-value 0.7317 | β 0.7552  SE 0.3187  CI lower 0.1307  CI upper 1.3798  t 2.3700  p-value 0.0419 | β 1.2485  SE 2.1728  CI lower -3.0102  CI upper 5.5072  t 0.5746  p-value 0.5703 | β -1.3430  SE 1.4498  CI lower -4.1846  CI upper 1.4985  t -0.9264  p-value 0.3619 | β -1.5934  SE 1.2129  CI lower -3.9707  CI upper 0.7839  t -1.3137  p-value 0.1993 | |
| *Body size* | β -5.1439  SE 5.2151  CI lower -15.3654  CI upper 5.0777  t -0.9863  p-value 0.3321 | β -0.5086  SE 1.4491  CI lower -3.3489  CI upper 2.3318  t -0.3509  p-value 0.7299 | β 3.1006  SE 1.2813  CI lower 0.5892  CI upper 5.6120  t 2.4199  p-value 0.0386 | β -1.2725  SE 5.6674  CI lower -12.3805  CI upper 9.8355  t -0.2245  p-value 0.8240 | β 4.9889  SE 3.9567  CI lower -2.7663  CI upper 12.7441  t 1.2609  p-value 0.2174 | β 2.3540  SE 3.3103  CI lower -4.1342  CI upper 8.8423  t 0.7111  p-value 0.4826 | |
| *Feeding behavior* | β -2.1941  SE 2.8196  CI lower -7.7205  CI upper 3.3323  t -0.7782  p-value 0.4427 | β -1.5689  SE 0.9833  CI lower -3.4961  CI upper 0.3584  t -1.5955  p-value 0.1290 | β 0.0419  SE 0.8656  CI lower -1.6547  CI upper 1.7386  t 0.0484  p-value 0.9624 | β 4.2289  SE 3.1659  CI lower -1.9836  CI upper 10.4415  t 1.3342  p-value 0.1932 | β 0.5850  SE 2.1392  CI lower -3.6079  CI upper 4.7779  t 0.2735  p-value 0.7864 | β 0.5566  SE 1.7898  CI lower -2.9513  CI upper 4.0646  t 0.3110  p-value 0.7580 | |
| *Environmental breadth* | β 3.6536  SE 1.9107  CI lower -0.0914  CI upper 1.9122  t 1.9122  p-value 0.0657 | β 1.0534  SE 1.6531  CI lower -2.1867  CI upper 4.2934  t 0.6372  p-value 0.5325 | β 0.0848  SE 0.3730  CI lower -0.6463  CI upper 0.8158  t 0.2272  p-value 0.8253 | β 5.1764  SE 2.0796  CI lower 1.1005  CI upper 9.2524  t 2.4892  p-value 0.0193 | β 5.1293  SE 1.4497  CI lower 2.2880  CI upper 7.9707  t 3.5383  p-value 0.0013 | β 5.7175  SE 1.2128  CI lower 3.3404  CI upper 8.0947  t 4.7141  p-value 0.0001 | |
| *Group size* | β -0.1209  SE 1.1804  CI lower -2.4345  CI upper 2.1928  t -0.1024  p-value 0.9191 | β -0.0361  SE 0.2198  CI lower -0.4669  CI upper 0.3947  t -0.1643  p-value 0.8714 | β 0.3544  SE 0.1818  CI lower -0.0020  CI upper 0.7108  t 1.9489  p-value 0.0831 | β 1.774  SE 1.2951  CI lower -0.7609  CI upper 4.3157  t 1.3724  p-value 0.1812 | β 0.1921  SE 0.8956  CI lower -1.5633  CI upper 1.9475  t 0.2145  p-value 0.8317 | β 0.3629  SE 0.7493  CI lower -1.1057  CI upper 1.8314  t 0.4844  p-value 0.6318 | |
| *Sociability* | β 2.7077  SE 1.2700  CI lower 0.2185  CI upper 5.1968  t 2.1320  p-value 0.0416 | β -0.1931  SE 0.2527  CI lower -0.6883  CI upper 0.3002  t -0.7641  p-value 0.4553 | β -0.6559  SE 0.1954  CI lower -1.0389  CI upper -0.2729  t -3.3566  p-value 0.0084 | β 1.9522  SE 1.4354  CI lower -0.8613  CI upper 4.7656  t 1.3600  p-value 0.1850 | β 2.3321  SE 0.9636  CI lower 0.4435  CI upper 4.2207  t 2.4203  p-value 0.0220 | β 1.6387  SE 0.8061  CI lower 0.0587  CI upper 3.2188  t 2.0328  p-value 0.0513 | |
| *Swarming* | β -1.3555  SE 1.9300  CI lower -5.1383  CI upper 2.4272  t -0.7024  p-value 0.4880 | β 0.4111  SE 0.3991  CI lower -0.3771  CI upper 1.1934  t 1.0301  p-value 0.3174 | β 0.6663  SE 0.2821  CI lower 0.1134  CI upper 1.2193  t 2.3619  p-value 0.0424 | β 0.4663  SE 2.1006  CI lower -3.6508  CI upper 4.5834  t 0.2220  p-value 0.8260 | β 0.5237  SE 1.4643  CI lower -2.3463  CI upper 3.3976  t 0.3576  p-value 0.7232 | β 0.7295  SE 1.2251  CI lower -1.6716  CI upper 3.1307  t 0.5955  p-value 0.5561 | |
| **df** | 29 | 17 | 9 | 27 | 29 | 29 | |
| **Shapiro-Wilk normality test** | W= 0.8547  p-value 0.0002 | W= 0.9581  p-value 0.3792 | W= 0.9739  p-value 0.8827 | W= 0.9589  p-value 0.2128 | W= 0.9429  p-value 0.0574 | W= 0.9405  p-value 0.0578 | |
| **BOXCOX** | | | | | | |  |
| *Lineage diversity* | β -0.0274  SE 0.5390  CI lower -1.0838  CI upper 1.0289  t -0.0509  p-value 0.9597 | / | / | / | / | / | |
| *Body size* | β -1.3227  SE 1.4709  CI lower -4.2057  CI upper 1.5603  t -0.8992  p-value 0.3759 | / | / | / | / | / | |
| *Feeding behavior* | β -0.4189  SE 0.7953  CI lower -1.9776  CI upper 1.1398  t -0.5267  p-value 0.6024 | / | / | / | / | / | |
| *Environmental breadth* | β 0.4930  SE 0.5389  CI lower -0.5633  CI upper 1.5492  t 0.9147  p-value 0.3679 | / | / | / | / | / | |
| *Group size* | β 0.1697  SE 0.3329  CI lower -0.4829  CI upper 0.8222  t 0.5095  p-value 0.6142 | / | / | / | / | / | |
| *Sociability* | β 0.4489  SE 0.3582  CI lower -0.2532  CI upper 1.1510  t 1.2533  p-value 0.2201 | / | / | / | / | / | |
| *Swarming* | β -0.0246  SE 0.5444  CI lower -1.0916  CI upper 1.0423  t -0.0453  p-value 0.9642 | / | / | / | / | / | |
| **df** | 29 |  |  |  |  |  | |
| **Shapiro-Wilk normality test** | W= 0.9562  p-value 0.1536 | / | / | / | / | / | |

**Figure S2. Temporal trend in the number of publications.** The annual number of studies included in the database over time.

| Virus | Number of publications |
| --- | --- |
| Adenoviridae | 17 |
| Papillomaviridae | 10 |
| Herpesviridae | 15 |
| Filoviridae | 14 |
| Lyssavirus | 103 |
| Coronaviridae | 48 |

**Figure S3.** Number of published studies for each viral group (*Adenoviridae*, *Papillomaviridae*, *Herpesviridae*, *Filoviridae*, *Lyssavirus*, and *Coronaviridae*) included in this study.

Temporality

| Years | Total number of publications |
| --- | --- |
| 1956 | 1 |
| 1992 | 3 |
| 1995 | 1 |
| 1996 | 1 |
| 1997 | 1 |
| 2000 | 1 |
| 2001 | 2 |
| 2002 | 1 |
| 2003 | 3 |
| 2004 | 2 |
| 2005 | 5 |
| 2006 | 5 |
| 2007 | 4 |
| 2008 | 6 |
| 2009 | 7 |
| 2010 | 6 |
| 2011 | 9 |
| 2012 | 6 |
| 2013 | 10 |
| 2014 | 11 |
| 2015 | 5 |
| 2016 | 4 |
| 2017 | 10 |
| 2018 | 10 |
| 2019 | 6 |
| 2020 | 7 |
| 2021 | 7 |
| 2022 | 11 |
| 2023 | 10 |

**Figure S4.** Temporal trend in the number of publications included in this study. Total number of studies collected per year across all viral groups.

**Figure S5.** **Correlations among host traits across viral groups.** Pairwise correlation coefficients were calculated among host traits (*lineage diversity*, *body* *size*, ***CM3***, *w****ing loading*,** *feeding behavior*, *environmental breadth*, *group size*, *sociability,* and ***sampling effort***) for each viral group.

**References**

Alkhovsky, S., Lenshin, S., Romashin, A., Vishnevskaya, T., Vyshemirsky, O., Bulycheva, Y., Lvov, D., Gitelman, A., 2022. SARS-Like Coronaviruses in Horseshoe Bats (*Rhinolophus* spp.) in Russia, 2020. Viruses 14. https://doi.org/10.3390/v14010113

Amengual, B., Bourhy, H., López-Roíg, M., Serra-Cobo, J., 2007. Temporal dynamics of European bat lyssavirus type 1 and survival of *Myotis myotis* bats in natural colonies. PLoS One 2, e566. https://doi.org/10.1371/journal.pone.0000566

Amengual, B., Whitby, J.E., King, A., Cobo, J.S., Bourhy, H., 1997. Evolution of European bat lyssaviruses. Journal of General Virology 78, 2319–2328.

Annan, A., Baldwin, H.J., Corman, V.M., Klose, S.M., Owusu, M., Nkrumah, E.E., Badu, E.K., Anti, P., Agbenyega, O., Meyer, B., Oppong, S., Sarkodie, Y.A., Kalko, E.K.V., Lina, P.H.C., Godlevska, E. V., Reusken, C., Seebens, A., Gloza-Rausch, F., Vallo, P., Tschapka, M., Drosten, C., Drexler, J.F., 2013. Human betacoronavirus 2c EMC/2012-related viruses in bats, Ghana and Europe. Emerg Infect Dis 19, 456–459. https://doi.org/10.3201/eid1903.121503

Ar Gouilh, M., Puechmaille, S.J., Diancourt, L., Vandenbogaert, M., Serra-Cobo, J., Lopez Roïg, M., Brown, P., Moutou, F., Caro, V., Vabret, A., Manuguerra, J.C., 2018. SARS-CoV related *Betacoronavirus* and diverse *Alphacoronavirus* members found in western old-world. Virology 517, 88–97. https://doi.org/10.1016/j.virol.2018.01.014

Arnaout, Y., Picard-Meyer, E., Robardet, E., Cappelle, J., Cliquet, F., Touzalin, F., Jimenez, G., Djelouadji, Z., 2023. Assessment of virus and *Leptospira* carriage in bats in France. PLoS One 18, e0292840. https://doi.org/10.1371/journal.pone.0292840

August, T.A., Mathews, F., Nunn, M.A., 2012. *Alphacoronavirus* detected in bats in the United Kingdom. Vector-Borne and Zoonotic Diseases 12, 530–533. https://doi.org/10.1089/vbz.2011.0829

Badrane, H., Bahloul, C., Perrin, P., Tordo, N., 2001. Evidence of Two *Lyssavirus* Phylogroups with Distinct Pathogenicity and Immunogenicity . J Virol 75, 3268–3276. https://doi.org/10.1128/jvi.75.7.3268-3276.2001

Balboni, A., Gallina, L., Palladini, A., Prosperi, S., Battilani, M., 2012. A real-time PCR assay for bat SARS-Like coronavirus detection and its application to Italian greater horseshoe bat faecal sample surveys. The Scientific World Journal. https://doi.org/10.1100/2012/989514

Balboni, A., Palladini, A., Bogliani, G., Battilani, M., 2011. Detection of a virus related to betacoronaviruses in Italian greater horseshoe bats. Epidemiol Infect 139, 216–219. https://doi.org/10.1017/S0950268810001147

Banyard, A.C., Johnson, N., Voller, K., Hicks, D., Nunez, A., Hartley, M., Fooks, A.R., 2009. Repeated detection of European bat lyssavirus type 2 in dead bats found at a single roost site in the UK. Arch Virol 154, 1847–1850. https://doi.org/10.1007/s00705-009-0504-8

Begeman, L., Kooi, E.A., van Weezep, E., van de Bildt, M.W.G., Reusken, C.B.E.M., Lina, P.H.C., Koopmans, M.P.G., van den Brand, J.M.A., Kuiken, T., 2020. Faeces as a novel material to estimate lyssavirus prevalence in bat populations. Zoonoses Public Health 67, 198–202. https://doi.org/10.1111/zph.12672

Botvinkin, A.D., Poleschuk, E.M., Kuzmin, I. V., Borisova, T.I., Gazaryan, S. V., Yager, P., Rupprecht, C.E., n.d. Novel lyssaviruses isolated from bats in Russia. Emerg Infect Dis 1623–1625.

Bourhy, H., Cowley, J.A., Larrous, F., Holmes, E.C., Walker, P.J., 2005. Phylogenetic relationships among rhabdoviruses inferred using the L polymerase gene. Journal of General Virology 86, 2849–2858. https://doi.org/10.1099/vir.0.81128-0

Bourhy, H., Kissi, B., Lafon, M., Sacramento, D., 1992. Antigenic and molecular characterization of bat rabies virus in Europe. J Clin Microbiol 30, 2419–2426.

Briestenská, K., Janíková, M., Kabát, P., Csepányiová, D., Zukal, J., Pikula, J., Kováčová, V., Linhart, P., Banďouchová, H., Mistríková, J., 2018a. Bats as another potential source of murine gammaherpesvirus 68 (MHV-68) in nature. Acta Virol 62, 337–339. https://doi.org/10.4149/av_2018_229

Briestenská, K., Janíková, M., Kabát, P., Csepányiová, D., Zukal, J., Pikula, J., Kováčová, V., Linhart, P., Banďouchová, H., Mistríková, J., 2018b. Bats as another potential source of murine gammaherpesvirus 68 (MHV-68) in nature. Acta Virol 62, 337–339. https://doi.org/10.4149/av_2018_229

Calvelage, S., Freuling, C.M., Fooks, A.R., Höper, D., Marston, D.A., McElhinney, L., Rasmussen, T.B., Finke, S., Beer, M., Müller, T., 2021. Full-genome sequences and phylogenetic analysis of archived danish European bat lyssavirus 1 (EBLV-1) emphasize a higher genetic resolution and spatial segregation for sublineage 1a. Viruses 13. https://doi.org/10.3390/v13040634

Ceballos, N.A., Morón, S.V., Berciano, J.M., Nicolás, O., López, C.A., Juste, J., Nevado, C.R., Setién, Á.A., Echevarría, J.E., 2013. Novel lyssavirus in bat, Spain. Emerg Infect Dis 19, 793–795. https://doi.org/10.3201/eid1905.121071

Černe, D., Hostnik, P., Toplak, I., Presetnik, P., Maurer-Wernig, J., Kuhar, U., 2023. Discovery of a novel bat lyssavirus in a Long-fingered bat (*Myotis capaccinii*) from Slovenia. PLoS Negl Trop Dis 17, e0011420. https://doi.org/10.1371/journal.pntd.0011420

Cholleti, H., de Jong, J., Blomström, A.L., Berg, M., 2023. Investigation of the Virome and Characterization of Issyk-Kul Virus from Swedish *Myotis brandtii* bats. Pathogens 12. https://doi.org/10.3390/pathogens12010012

Cholleti, H.;, De Jong, J.;, Blomström, A.-L.;, Berg, M., 2022. Characterization of *Pipistrellus pygmaeus* bat virome from Sweden. Palliat Med 14. https://doi.org/10.3390/v14081654

Dacheux, L., Cervantes-Gonzalez, M., Guigon, G., Thiberge, J.M., Vandenbogaert, M., Maufrais, C., Caro, V., Bourhy, H., 2014. A preliminary study of viral metagenomics of french bat species in contact with humans: Identification of new mammalian viruses. PLoS One 9. https://doi.org/10.1371/journal.pone.0087194

Dacheux, L., Larrous, F., Mailles, A., Boisseleau, D., Delmas, O., Biron, C., Bouchier, C., Capek, I., Muller, M., Ilari, F., Lefranc, T., Raffi, F., Goudal, M., Bourhy, H., 2009. European bat lyssavirus transmission among cats, Europe. Emerg Infect Dis 15, 280–284. https://doi.org/10.3201/eid1502.080637

Davis, P.L., Holmes, E.C., Larrous, F., Van der Poel, W.H.M., Tjørnehøj, K., Alonso, W.J., Bourhy, H., 2005. Phylogeography, population dynamics, and molecular evolution of European bat lyssaviruses. J Virol 79, 10487–10497. https://doi.org/10.1128/jvi.79.16.10487-10497.2005

De Arellano, E.R., Sanchez-Lockhart, M., Perteguer, M.J., Bartlett, M., Ortiz, M., Campioli, P., Hernández, A., Gonzalez, J., Garcia, K., Ramos, M., Jiménez-Clavero, M.Á., Tenorio, A., Sánchez-Seco, M.P., González, F., Echevarría, J.E., Palacios, G., Negred, A., 2019. First evidence of antibodies against Lloviu virus in schreiber’s bent-winged insectivorous bats demonstrate a wide circulation of the virus in spain. Viruses 11. https://doi.org/10.3390/v11040360

De Benedictis, P., Marciano, S., Scaravelli, D., Priori, P., Zecchin, B., Capua, I., Monne, I., Cattoli, G., 2014. Alpha and lineage C betaCoV infections in Italian bats. Virus Genes 48, 366–371. https://doi.org/10.1007/s11262-013-1008-x

De Sabato, L., Lelli, D., Faccin, F., Canziani, S., Di Bartolo, I., Vaccari, G., Moreno, A., 2019. Full genome characterization of two novel Alpha-coronavirus species from Italian bats. Virus Res 260, 60–66. https://doi.org/10.1016/j.virusres.2018.11.007

Delbos, V., Abgueguen, P., Chennebault, J.-M., Pichard, E., 2006. Un nouveau cas de rage chez les chauves-souris en France. Revue de Medecine Interne 27, 575–577. https://doi.org/10.1016/j.revmed.2006.04.002

Delmas, O., Holmes, E.C., Talbi, C., Larrous, F., Dacheux, L., Bouchier, C., Bourhy, H., 2008. Genomic diversity and evolution of the lyssaviruses. PLoS One 3, e2057. https://doi.org/10.1371/journal.pone.0002057

Diakoudi, G., Lanave, G., Moreno, A., Chiapponi, C., Sozzi, E., Prosperi, A., Larocca, V., Losurdo, M., Decaro, N., Martella, V., Lavazza, A., Lelli, D., 2019. Surveillance for adenoviruses in bats in Italy. Viruses 11. https://doi.org/10.3390/v11060523

Dietz, C., Kiefer, A., 2017. Murciélagos de Europa: conocerlos, identificarlos y protegerlos, Omega. ed. Barcelona.

Drexler, J.F., Corman, V.M., Wegner, T., Tateno, A.F., Zerbinati, R.M., Gloza-Rausch, F., Seebens, A., Müller, M.A., Drosten, C., 2011. Amplification of emerging viruses in a bat colony. Emerg Infect Dis 17, 449–456. https://doi.org/10.3201/eid1703.100526

Drexler, J.F., Gloza-Rausch, F., Glende, J., Corman, V.M., Muth, D., Goettsche, M., Seebens, A., Niedrig, M., Pfefferle, S., Yordanov, S., Zhelyazkov, L., Hermanns, U., Vallo, P., Lukashev, A., Müller, M.A., Deng, H., Herrler, G., Drosten, C., 2010. Genomic characterization of severe acute respiratory syndrome-related coronavirus in European bats and classification of coronaviruses based on partial RNA-dependent RNA polymerase gene sequences. J Virol 84, 11336–11349. https://doi.org/10.1128/jvi.00650-10

Falcón, A., Vázquez-Morón, S., Casas, I., Aznar, C., Ruiz, G., Pozo, F., Perez-Breña, P., Juste, J., Ibáñez, C., Garin, I., Aihartza, J., Echevarría, J.E., 2011. Detection of alpha and betacoronaviruses in multiple Iberian bat species. Arch Virol 156, 1883–1890. https://doi.org/10.1007/s00705-011-1057-1

Fischer, K., Zeus, V., Kwasnitschka, L., Kerth, G., Haase, M., Groschup, M.H., Balkema-Buschmann, A., 2016. Insectivorous bats carry host specific astroviruses and coronaviruses across different regions in Germany. Infection, Genetics and Evolution 37, 108–116. https://doi.org/10.1016/j.meegid.2015.11.010

Folly, A.J., Marston, D.A., Golding, M., Shukla, S., Wilkie, R., Lean, F.Z.X., Núñez, A., Worledge, L., Aegerter, J., Banyard, A.C., Fooks, A.R., Johnson, N., McElhinney, L.M., 2021. Incursion of European bat lyssavirus 1 (Eblv-1) in serotine bats in the United Kingdom. Viruses 13. https://doi.org/10.3390/v13101979

Fooks, A.R., Marston, D., Parsons, G., Earl, D., Dicker, A., Brookes, S.M., 2006. Isolation of EBLV-2 in a Daubenton’s bat (*Myotis daubentonii*) found in Oxfordshire. Vet Rec 534–535.

Fooks, A.R., McElhinney, L.M., Pounder, D.J., Finnegan, C.J., Mansfield, K., Johnson, N., Brookes, S.M., Parsons, G., White, K., McIntyre, P.G., Nathwanis, D., 2003. Case report: Isolation of a European bat lyssavirus type 2a from a fatal human case of rabies encephalitis. J Med Virol 71, 281–289. https://doi.org/10.1002/jmv.10481

Freuling, C., Grossmann, E., Conraths, F.J., Schameitat, A., Kliemt, J., Auer, E., Greiser-Wilke, I., Müller, T., 2008. First Isolation of EBLV-2 in Germany. Vet Microbiol 131, 26–34. https://doi.org/10.1016/j.vetmic.2008.02.028

Freuling, C.M., Abendroth, B., Beer, M., Fischer, M., Hanke, D., Hoffmann, B., Höper, D., Just, F., Mettenleiter, T.C., Schatz, J., Müller, T., 2013. Molecular diagnostics for the detection of Bokeloh bat lyssavirus in a bat from Bavaria, Germany. Virus Res 177, 201–204. https://doi.org/10.1016/j.virusres.2013.07.021

Freuling, C.M., Beer, M., Conraths, F.J., Finke, S., Hoffmann, B., Keller, B., Kliemt, J., Mettenleiter, T.C., Mühlbach, E., Teifke, J.P., Wohlsein, P., Müller, T., 2011. Novel lyssavirus in Natterer’s bat, Germany. Emerg Infect Dis 17, 1519–1522. https://doi.org/10.3201/eid1708.110201

Freuling, C.M., Hoffmann, B., Selhorst, T., Conraths, F.J., Kliemt, J., Schatz, J., Müller, T., 2012. New insights into the genetics of EBLV-1 from Germany. Berl Münch Tierärztl Wochenschr 125, 255–263. https://doi.org/10.2376/0005-5366-125-255

Froidevaux, J. S. P., Toshkova, N., Barbaro, L., Benítez-López, A., Kerbiriou, C., Le Viol, I., Pacifici, M., Santini, L., Stawski, C., Russo, D., Dekker, J., Alberdi, A., Amorim, F., Ancillotto, L., Barré, K., Bas, Y., Cantú-Salazar, L., Dechmann, D. K. N., Devaux, T., … Razgour, O. (2023). A species-level trait dataset of bats in Europe and beyond. Scientific Data, 10(253). https://doi.org/10.1038/s41597-023-02157-4

García-Pérez, R., Ibáñez, C., Godínez, J.M., Aréchiga, N., Garin, I., Pérez-Suárez, G., De Paz, O., Juste, J., Echevarría, J.E., Bravo, I.G., 2014. Novel papillomaviruses in free-ranging Iberian bats: No virus-host co-evolution, no strict host specificity, and hints for recombination. Genome Biol Evol 6, 94–104. https://doi.org/10.1093/gbe/evt211

Gloza-Rausch, F., Ipsen, A., Seebens, A., Göttsche, M., Panning, M., Drexler, J.F., Petersen, N., Annan, A., Grywna, K., Müller, M., Pfefferle, S., Drosten, C., 2008. Detection and prevalence patterns of group I coronaviruses in bats, northern Germany. Emerg Infect Dis 14, 626–631.

Goffard, A., Demanche, C., Arthur, L., Pinçon, C., Michaux, J., Dubuisson, J., 2015. Alphacoronaviruses detected in french bats are phylogeographically linked to coronaviruses of european bats. Viruses 7, 6279–6290. https://doi.org/10.3390/v7122937

Hackländer, K., & Zachos, F. E. (2023). Handbook of the Mammals of Europe. Chiroptera (Danilo Russo, Ed.; Springer).

Hardmeier, I., Aeberhard, N., Qi, W., Schoenbaechler, K., Kraettli, H., Hatt, J.M., Fraefel, C., Kubacki, J., 2021. Metagenomic analysis of fecal and tissue samples from 18 endemic bat species in Switzerland revealed a diverse virus composition including potentially zoonotic viruses. PLoS One 16, e0252534. https://doi.org/10.1371/journal.pone.0252534

Harris, S.L., Aegerter, J.N., Brookes, S.M., Mcelhinney, L.M., Jones, G., Smith, G.C., Fooks, A.R., 2009. Targeted surveillance for European bat lyssaviruses in English bats (2003-06). J Wildl Dis 45, 1030–1041.

Harris, S.L., Mansfield, K., Marston, D.A., Johnson, N., Pajamo, K., O’Brien, N., Black, C., Mcelhinney, L.M., Fooks, A.R., 2007. Isolation of European bat lyssavirus type 2 from a Daubenton’s bat (*Myotis daubentonii*) in Shropshire. Veterinary Record. https://doi.org/10.1136/vr.161.11.384

Hemnani, M., Silva, P.G. da, Thompson, G., Poeta, P., Rebelo, H., Mesquita, J.R., 2023. First report of *Alphacoronavirus* circulating in cavernicolous bats from Portugal. Viruses 15. https://doi.org/10.3390/v15071521

Horton, D.L., Breed, A.C., Arnold, M.E., Smith, G.C., Aegerter, J.N., McElhinney, L.M., Johnson, N., Banyard, A.C., Raynor, R., Mackie, I., Denwood, M.J., Mellor, D.J., Swift, S., Racey, P.A., Fooks, A.R., 2020. Between roost contact is essential for maintenance of European bat lyssavirus type-2 in *Myotis daubentonii* bat reservoir: ‘The Swarming Hypothesis.’ Sci Rep 10. https://doi.org/10.1038/s41598-020-58521-6

Iglesias-Caballero, M., Juste, J., Vázquez-Morón, S., Falcon, A., Aznar-Lopez, C., Ibáñez, C., Pozo, F., Ruiz, G., Berciano, J.M., Garin, I., Aihartza, J., Echevarría, J.E., Casas, I., 2018. New adenovirus groups in western palaearctic bats. Viruses 10. https://doi.org/10.3390/v10080443

IUCN. (2025, April). The IUCN Red List of Threatened Species. Version 2025-1.

Jakava-Viljanen, M., Lilley, T., Kyheröinen, E.M., Huovilainen, A., 2010. First encounter of European bat lyssavirus type 2 (EBLV-2) in a bat in Finland. Epidemiol Infect 138, 1581–1585. https://doi.org/10.1017/S0950268810000373

Jánoska, M., Vidovszky, M., Molnár, V., Liptovszky, M., Harrach, B., Benko, M., 2011. Novel adenoviruses and herpesviruses detected in bats. Veterinary Journal 189, 118–121. https://doi.org/10.1016/j.tvjl.2010.06.020

Johnson, N., Selden, D., Parsons, G., Healy, D., Brookes, S.M., McElhinney, L.M., Hutson, A.M., Fooks, A.R., 2003. Isolation of a European bat lyssavirus type 2 from a Daubenton’s bat in the United Kingdom. Veterinary Record 152, 383–387. https://doi.org/10.1136/vr.152.13.383

Johnson, N., Wakeley, P.R., Brookes, S.M., Fooks, A.R., 2006. European bat lyssavirus type 2 RNA in *Myotis daubentonii*. Emerg Infect Dis 12, 1142–1144. https://doi.org/10.3201/eid1207.060287

Juste, J., Ruedi, M., Puechmaille, S. J., Salicini, I., & Ibáñez, C. (2018). Two New Cryptic Bat Species within the *Myotis nattereri* Species Complex (Vespertilionidae, Chiroptera) from the Western Palaearctic. Acta Chiropterologica, 20(2), 285–300. https://doi.org/10.3161/15081109ACC2018.20.2.001

Kappeier Andreas, 1992. First Case of Rabies in a Bat in Switzerland. Veterinary Bacteriological Institute 11.

Kemenesi, G., Dallos, B., Görföl, T., Boldogh, S., Estók, P., Kurucz, K., Kutas, A., Földes, F., Oldal, M., Németh, V., Martella, V., Bányai, K., Jakab, F., 2014. Molecular survey of RNA viruses in hungarian bats: Discovering novel astroviruses, coronaviruses, and caliciviruses. Vector-Borne and Zoonotic Diseases 14, 846–855. https://doi.org/10.1089/vbz.2014.1637

Kemenesi, G., Kurucz, K., Dallos, B., Zana, B., Földes, F., Boldogh, S., Görföl, T., Carroll, M.W., Jakab, F., 2018. Re-emergence of Lloviu virus in *Miniopterus schreibersii* bats, Hungary, 2016. Emerg Microbes Infect 7. https://doi.org/10.1038/s41426-018-0067-4

Kemenesi, G., Tóth, G.E., Mayora-Neto, M., Scott, S., Temperton, N., Wright, E., Mühlberger, E., Hume, A.J., Suder, E.L., Zana, B., Boldogh, S.A., Görföl, T., Estók, P., Lanszki, Z., Somogyi, B.A., Nagy, Á., Pereszlényi, C.I., Dudás, G., Földes, F., Kurucz, K., Madai, M., Zeghbib, S., Maes, P., Vanmechelen, B., Jakab, F., 2022. Isolation of infectious Lloviu virus from Schreiber’s bats in Hungary. Nat Commun 13. https://doi.org/10.1038/s41467-022-29298-1

Kivistö, I., Tidenberg, E.M., Lilley, T., Suominen, K., Forbes, K.M., Vapalahti, O., Huovilainen, A., Sironen, T., 2020. First report of coronaviruses in northern European bats. Vector-Borne and Zoonotic Diseases 20, 155–158. https://doi.org/10.1089/vbz.2018.2367

Klein, A., Calvelage, S., Schlottau, K., Hoffmann, B., Eggerbauer, E., Müller, T., Freuling, C.M., 2021. Retrospective enhanced bat lyssavirus surveillance in Germany between 2018–2020. Viruses 13. https://doi.org/10.3390/v13081538

Kohl, C., Brinkmann, A., Radonić, A., Dabrowski, P.W., Mühldorfer, K., Nitsche, A., Wibbelt, G., Kurth, A., 2021. The virome of German bats: comparing virus discovery approaches. Sci Rep 11. https://doi.org/10.1038/s41598-021-86435-4

Kohl, C., Vidovszky, M.Z., Mühldorfer, K., Dabrowski, P.W., Radonić, A., Nitsche, A., Wibbelt, G., Kurth, A., Harrach, B., 2012. Genome analysis of bat adenovirus 2: indications of interspecies transmission. J Virol 86, 1888–1892. https://doi.org/10.1128/jvi.05974-11

Korytár, Ľ., Ondrejková, A., Drážovská, M., Zemanová, S., Prokeš, M., 2022. Serological survey of lyssaviruses in synanthropic bats and human exposure to bats in Slovakia. Annals of Agricultural and Environmental Medicine 29, 44–49. https://doi.org/10.26444/aaem/146208

Kuzmin, I. V., Hughes, G.J., Botvinkin, A.D., Orciari, L.A., Rupprecht, C.E., 2005. Phylogenetic relationships of Irkut and West Caucasian bat viruses within the *Lyssavirus* genus and suggested quantitative criteria based on the N gene sequence for lyssavirus genotype definition. Virus Res 111, 28–43. https://doi.org/10.1016/j.virusres.2005.03.008

Lazov, C.M., Belsham, G.J., Bøtner, A., Rasmussen, T.B., 2021. Full-genome sequences of alphacoronaviruses and astroviruses from myotis and pipistrelle bats in Denmark. Viruses 13. https://doi.org/10.3390/v13061073

Lazov, C.M., Chriél, M., Baagøe, H.J., Fjederholt, E., Deng, Y., Kooi, E.A., Belsham, G.J., Bøtner, A., Rasmussen, T.B., 2018. Detection and characterization of distinct alphacoronaviruses in five different bat species in Denmark. Viruses 10. https://doi.org/10.3390/v10090486

Lecis, R., Mucedda, M., Pidinchedda, E., Pittau, M., Alberti, A., 2019. Molecular identification of *Betacoronavirus* in bats from Sardinia (Italy): first detection and phylogeny. Virus Genes 55, 60–67. https://doi.org/10.1007/s11262-018-1614-8

Lelli, D., Papetti, A., Sabelli, C., Rosti, E., Moreno, A., Boniotti, M.B., 2013. Detection of coronaviruses in bats of various species in Italy. Viruses 5, 2679–2689. https://doi.org/10.3390/v5112679

Leopardi, S., Desiato, R., Mazzucato, M., Orusa, R., Obber, F., Averaimo, D., Berjaoui, S., Canziani, S., Capucchio, M.T., Conti, R., Di Bella, S., Festa, F., Garofalo, L., Lelli, D., Madrau, M.P., Mandola, M.L., Moreno Martin, A.M., Peletto, S., Pirani, S., Robetto, S., Torresi, C., Varotto, M., Citterio, C., Terregino, C., 2023. One health surveillance strategy for coronaviruses in Italian wildlife. Epidemiol Infect 151, 1–10. https://doi.org/10.1017/S095026882300081X

López-Roig, M., Bourhy, H., Lavenir, R., Serra-Cobo, J., 2014. Seroprevalence dynamics of European bat Lyssavirus type 1 in a multispecies bat colony. Viruses 6, 3386–3399. https://doi.org/10.3390/v6093386

Lwande, O.W., Thalin, T., de Jong, J., Sjödin, A., Näslund, J., Evander, M., Ecke, F., 2022. *Alphacoronavirus* in a Daubenton’s Myotis Bat (*Myotis daubentonii*) in Sweden. Viruses 14. https://doi.org/10.3390/v14030556

Marston, D.A., McElhinney, L.M., Ellis, R.J., Horton, D.L., Wise, E.L., Leech, S.L., David, D., de Lamballerie, X., Fooks, A.R., 2013. Next generation sequencing of viral RNA genomes. BMC Genomics 14, 444. https://doi.org/10.1186/1471-2164-14-444

Marston, D.A., McElhinney, L.M., Johnson, N., Müller, T., Conzelmann, K.K., Tordo, N., Fooks, A.R., 2007. Comparative analysis of the full genome sequence of *European bat lyssavirus type* 1 and type 2 with other lyssaviruses and evidence for a conserved transcription termination and polyadenylation motif in the G-L 3′ non-translated region. Journal of General Virology 88, 1302–1314. https://doi.org/10.1099/vir.0.82692-0

Marston, D.A., Vázquez-Morón, S., Ellis, R.J., Wise, E.L., McElhinney, L.M., de Lamballerie, X., Fooks, A.R., Echevarría, J.E., 2015. Complete genomic sequence of European bat lyssavirus 1, isolated from *Eptesicus isabellinus* in Spain. Genome Announc 3. https://doi.org/10.1128/genomeA.01518-14

Miia, J.V., Tiina, N., Tarja, S., Olli, V., Liisa, S., Anita, H., 2015. Evolutionary trends of *European bat lyssavirus type 2* including genetic characterization of Finnish strains of human and bat origin 24 years apart. Arch Virol 160, 1489–1498. https://doi.org/10.1007/s00705-015-2424-0

Mingo-Casas, P., Sandonís, V., Obón, E., Berciano, J.M., Vázquez-Morón, S., Juste, J., Echevarría, J.E., 2018. First cases of *European bat lyssavirus type 1* in Iberian serotine bats: Implications for the molecular epidemiology of bat rabies in Europe. PLoS Negl Trop Dis 12, e0006290. https://doi.org/10.1371/journal.pntd.0006290

Moldal, T., Vikøren, T., Cliquet, F., Marston, D.A., van der Kooij, J., Madslien, K., Ørpetveit, I., 2017. First detection of *European bat lyssavirus type 2* (EBLV-2) in Norway. BMC Vet Res 13. https://doi.org/10.1186/s12917-017-1135-z

Molnár, V., Jánoska, M., Harrach, B., Glávits, R., Pálmai, N., Rigó, D., Sós, E., Liptovszky, M., 2008. Detection of a novel bat gammaherpesvirus in Hungary. Acta Vet Hung 56, 529–538. https://doi.org/10.1556/AVet.56.2008.4.10

Monchatre-Leroy, E., Boué, F., Boucher, J.M., Renault, C., Moutou, F., Gouilh, M.A., Umhang, G., 2017. Identification of alpha and beta coronavirus in wildlife species in france: bats, rodents, rabbits, and hedgehogs. Viruses 9. https://doi.org/10.3390/v9120364

Moraga-Fernández, A., Sánchez-Sánchez, M., Queirós, J., Lopes, A.M., Vicente, J., Pardavila, X., Sereno-Cadierno, J., Alves, P.C., de la Fuente, J., Fernández de Mera, I.G., 2022. A study of viral pathogens in bat species in the Iberian Peninsula: identification of new coronavirus genetic variants. Int J Vet Sci Med 10, 100–110. https://doi.org/10.1080/23144599.2022.2139985

Moreno, A., Lelli, D., De Sabato, L., Zaccaria, G., Boni, A., Sozzi, E., Prosperi, A., Lavazza, A., Cella, E., Castrucci, M.R., Cicozzi, M., Vaccari, G., 2017. Detection and full genome characterization of two beta CoV viruses related to Middle East respiratory syndrome from bats in Italy. Virol J 14. https://doi.org/10.1186/s12985-017-0907-1

Mühldorfer, K., Speck, S., Kurth, A., Lesnik, R., Freuling, C., Müller, T., Kramer-Schadt, S., Wibbelt, G., 2011. Diseases and causes of death in European bats: Dynamics in disease susceptibility and infection rates. PLoS One 6. https://doi.org/10.1371/journal.pone.0029773

Müller, T., Johnson, N., Freuling, C.M., Fooks, A.R., Selhorst, T., Vos, A., 2007. Epidemiology of bat rabies in Germany. Arch Virol 152, 273–288. https://doi.org/10.1007/s00705-006-0853-5

Negredo, A., Palacios, G., Vázquez-Morón, S., González, F., Dopazo, H., Molero, F., Juste, J., Quetglas, J., Savji, N., de la Cruz Martínez, M., Herrera, J.E., Pizarro, M., Hutchison, S.K., Echevarría, J.E., Lipkin, W.I., Tenorio, A., 2011. Discovery of an ebolavirus-like filovirus in europe. PLoS Pathog 7. https://doi.org/10.1371/journal.ppat.1002304

Nieuwenhuijs, J., Haagsma, J., Lina, P., 1992. Epidemiology and control of rabies in bats in the Netherlands. Scientifique Et Technique-office International Des Epizooties 11, 1155–1161.

Nikolić, M., Jelesić, Z.M.D., 1956. Isolation of rabies virus from insectivorous bats in Yugoslavia. Bull World Health Organ 14, 801.

Nokireki, T., Huovilainen, A., Lilley, T., Kyheröinen, E.M., Ek-Kommonen, C., Sihvonen, L., Jakava-Viljanen, M., 2013. Bat rabies surveillance in Finland. BMC Vet Res 9. https://doi.org/10.1186/1746-6148-9-174

Nokireki, T., Sironen, T., Smura, T., Karkamo, V., Sihvonen, L., Gadd, T., 2017. Second case of *European bat lyssavirus type 2* detected in a Daubenton’s bat in Finland. Acta Vet Scand 59. https://doi.org/10.1186/s13028-017-0331-y

Nokireki, T., Tammiranta, N., Kokkonen, U.M., Kantala, T., Gadd, T., 2018. Tentative novel lyssavirus in a bat in Finland. Transbound Emerg Dis 65, 593–596. https://doi.org/10.1111/tbed.12833

Ondrejková, A., Franka, R., Ondrejka, R., Švrcek, Š., Süli, J., Benísek, Z., Zubrický, P., Bajova, V., Bourhy, H., Bugarský, A., 2004. Identification of a lyssavirus isolate from a bat (*Eptesicus serotinus*). Bulletin Veterinary Institute in Pulawy 48, 11–13.

Orłowska, A., Smreczak, M., Thor, K., Niedbalska, M., Pawelec, D., Trębas, P., Rola, J., 2022. The genetic characterization of the first detected bat coronaviruses in Poland revealed SARS-related types and alphacoronaviruses. Viruses 14. https://doi.org/10.3390/v14091914

Ortiz, L.J., Begeman, L., Schillemans, M., Kuiken, T., De Boer, W.F., 2023. Presence of coronaviruses in the common pipistrelle (*P. pipistrellus*) and Nathusiuś pipistrelle (*P. nathusii*) in relation to landscape composition. PLoS One 18, e0293649. https://doi.org/10.1371/journal.pone.0293649

Parize, P., Travecedo Robledo, I.C., Cervantes-Gonzalez, M., Kergoat, L., Larrous, F., Serra-Cobo, J., Dacheux, L., Bourhy, H., 2020. Circumstances of Human–Bat interactions and risk of lyssavirus transmission in metropolitan France. Zoonoses Public Health 67, 774–784. https://doi.org/10.1111/zph.12747

Pérez-Jordá, J.L., Ibáñez, C., Muñoz-Cervera, M., Téllez, A., 1995. *Lyssavirus* in *Eptesicus serotinus* (Chiroptera: Vespertilionidae). J Wildl Dis 31, 372–377.

Picard-Meyer, E., Barrat, J., Tissot, E., Barrat, M.J., Bruyére, V., Cliquet, F., 2004. Genetic analysis of *European bat lyssavirus type 1* isolates from France. Veterinary Record 154, 589–595. https://doi.org/10.1136/vr.154.19.589

Picard-Meyer, E., Beven, V., Hirchaud, E., Guillaume, C., Larcher, G., Robardet, E., Servat, A., Blanchard, Y., Cliquet, F., 2019. Lleida Bat Lyssavirus isolation in *Miniopterus schreibersii* in France. Zoonoses Public Health 66, 254–258. https://doi.org/10.1111/zph.12535

Picard-Meyer, E., Dubourg-Savage, M.J., Arthur, L., Barataud, M., Bécu, D., Bracco, S., Borel, C., Larcher, G., Meme-Lafond, B., Moinet, M., Robardet, E., Wasniewski, M., Cliquet, F., 2011. Active surveillance of bat rabies in France: A 5-year study (2004-2009). Vet Microbiol 151, 390–395. https://doi.org/10.1016/j.vetmic.2011.03.034

Picard-Meyer, E., Robardet, E., Arthur, L., Larcher, G., Harbusch, C., Servat, A., Cliquet, F., 2014. Bat rabies in France: A 24-year retrospective epidemiological study. PLoS One 9, e98622. https://doi.org/10.1371/journal.pone.0098622

Picard-Meyer, E., Servat, A., Robardet, E., Moinet, M., Borel, C., Cliquet, F., 2013. Isolation of Bokeloh bat lyssavirus in *Myotis nattereri* in France. Arch Virol 158, 2333–2340. https://doi.org/10.1007/s00705-013-1747-y

Picard-Meyer, E., Servat, A., Wasniewski, M., Gaillard, M., Borel, C., Cliquet, F., 2017. Bat rabies surveillance in France: First report of unusual mortality among serotine bats. BMC Vet Res 13. https://doi.org/10.1186/s12917-017-1303-1

Popov, Igor V., Ohlopkova, O. V., Donnik, I.M., Zolotukhin, P. V., Umanets, A., Golovin, S.N., Malinovkin, A. V., Belanova, A.A., Lipilkin, P. V., Lipilkina, T.A., Popov, Ilya V., Logvinov, A.K., Dubovitsky, N.A., Stolbunova, K.A., Sobolev, I.A., Alekseev, A.Y., Shestopalov, A.M., Burkova, V.N., Chikindas, M.L., Venema, K., Ermakov, A.M., 2023. Detection of coronaviruses in insectivorous bats of Fore-Caucasus, 2021. Sci Rep 13. https://doi.org/10.1038/s41598-023-29099-6

Pozo, F., Juste, J., Vázquez-Morón, S., Aznar-López, C., Ibáñez, C., Garin, I., Aihartza, J., Casas, I., Tenorio, A., Echevarría, J.E., 2016. Identification of novel betaherpesviruses in iberian bats reveals parallel evolution. PLoS One 11. https://doi.org/10.1371/journal.pone.0169153

Reusken, C.B.E.M., Lina, P.H.C., Pielaat, A., De Vries, A., Dam-Deisz, C., Adema, J., Drexler, J.F., Drosten, C., Kooi, E.A., 2010. Circulation of group 2 coronaviruses in a bat species common to urban areas in western Europe. Vector-Borne and Zoonotic Diseases 10, 785–791. https://doi.org/10.1089/vbz.2009.0173

Rihtarič, D., Hostnik, P., Steyer, A., Grom, J., Toplak, I., 2010. Identification of SARS-like coronaviruses in horseshoe bats (*Rhinolophus hipposideros*) in Slovenia. Arch Virol 155, 507–514. https://doi.org/10.1007/s00705-010-0612-5

Rizzo, F., Edenborough, K.M., Toffoli, R., Culasso, P., Zoppi, S., Dondo, A., Robetto, S., Rosati, S., Lander, A., Kurth, A., Orusa, R., Bertolotti, L., Mandola, M.L., 2017. Coronavirus and paramyxovirus in bats from Northwest Italy. BMC Vet Res 13. https://doi.org/10.1186/s12917-017-1307-x

Robardet, E., Borel, C., Moinet, M., Jouan, D., Wasniewski, M., Barrat, J., Boué, F., Montchâtre-Leroy, E., Servat, A., Gimenez, O., Cliquet, F., Picard-Meyer, E., 2017. Longitudinal survey of two serotine bat (*Eptesicus serotinus*) maternity colonies exposed to EBLV-1 (European Bat Lyssavirus type 1): Assessment of survival and serological status variations using capture-recapture models. PLoS Negl Trop Dis 11. https://doi.org/10.1371/journal.pntd.0006048

Rossetto, F., Iglesias-Caballero, M., Liedtke, H. C., Gomez-Mestre, I., Berciano, J. M., Pérez-Suárez, G., De Paz, O., Ibáñez, C., Echevarría, J. E., Casas, I., & Juste, J. (2020). Mating strategy is determinant of adenovirus prevalence in European bats. PLoS ONE, 15. https://doi.org/10.1371/journal.pone.0226203

Sander, A.-L., Moreira-Soto, A., Yordanov, S., Toplak, I., Balboni, A., Ameneiros, R.S., Corman, V., Drosten, C., Drexler, J.F., 2022. Genomic determinants of Furin cleavage in diverse European SARS-related bat coronaviruses. Commun Biol 5. https://doi.org/10.1038/s42003-022-03421-w

Schatz, Juliane, Freuling, C.M., Auer, E., Goharriz, H., Harbusch, C., Johnson, N., Kaipf, I., Mettenleiter, T.C., Mühldorfer, K., Mühle, R.U., Ohlendorf, B., Pott-Dörfer, B., Prüger, J., Ali, H.S., Stiefel, D., Teubner, J., Ulrich, R.G., Wibbelt, G., Müller, T., 2014. Enhanced passive bat rabies surveillance in indigenous bat species from Germany - a retrospective study. PLoS Negl Trop Dis 8, e2835. https://doi.org/10.1371/journal.pntd.0002835

Schatz, J., Ohlendorf, B., Busse, P., Pelz, G., Dolch, D., Teubner, J., Encarnação, J.A., Mühle, R.U., Fischer, M., Hoffmann, B., Kwasnitschka, L., Balkema-Buschmann, A., Mettenleiter, T.C., Müller, T., Freuling, C.M., 2014a. Twenty years of active bat rabies surveillance in Germany: A detailed analysis and future perspectives. Epidemiol Infect 142, 1155–1166. https://doi.org/10.1017/S0950268813002185

Schatz, J., Teifke, J.P., Mettenleiter, T.C., Aue, A., Stiefel, D., Müller, T., Freuling, C.M., 2014b. Lyssavirus distribution in naturally infected bats from Germany. Vet Microbiol 169, 33–41. https://doi.org/10.1016/j.vetmic.2013.12.004

Serra-Cobo, J., Amengual, B., Abellán, C., Bourhy, H., 2002. European Bat Lyssavirus Infection in Spanish Bat Populations. Emerg Infect Dis 8.

Serra-Cobo, J., López-Roig, M., Seguí, M., Sánchez, L.P., Nadal, J., Borrás, M., Lavenir, R., Bourhy, H., 2013. Ecological factors associated with *European Bat Lyssavirus* Seroprevalence in Spanish bats. PLoS One 8, e64467. https://doi.org/10.1371/journal.pone.0064467

Servat, A., Herr, J., Picard-Meyer, E., Schley, L., Harbusch, C., Michaux, C., Pir, J., Robardet, E., Engel, E., Cliquet, F., 2015. First isolation of a rabid bat infected with European bat lyssavirus in Luxembourg. Zoonoses Public Health 62, 7–10. https://doi.org/10.1111/zph.12095

Šimić, I., Lojkić, I., Krešić, N., Cliquet, F., Picard-Meyer, E., Wasniewski, M., Ćukušić, A., Zrnčić, V., Bedeković, T., 2018. Molecular and serological survey of lyssaviruses in Croatian bat populations. BMC Vet Res 14. https://doi.org/10.1186/s12917-018-1592-z

Šimić, I., Zorec, T.M., Lojkić, I., Krešić, N., Poljak, M., Cliquet, F., Picard-Meyer, E., Wasniewski, M., Zrnčić, V., Ćukušić, A., Bedeković, T., 2020. Viral metagenomic profiling of croatian bat population reveals sample and habitat dependent diversity. Viruses 12. https://doi.org/10.3390/v12080891

Smreczak, M., Orłowska, A., Marzec, A., Trębas, P., Müller, T., Freuling, C.M., Żmudziński, J.F., 2018. Bokeloh bat lyssavirus isolation in a Natterer’s bat, Poland. Zoonoses Public Health 65, 1015–1019. https://doi.org/10.1111/zph.12519

Smreczak, M., Orlowska, A., Trebas, P., 2020. Detection of BBLV in Natterer’s bat in Poland. Med Weter 76, 119–123. https://doi.org/10.21521/mw.6328

Smreczak, M., Orlowska, A., Zmudzinski, J.F., 2009. First case of the European bat Lyssavirus type 1B in bats (*Eptesicus serotinus*) in Poland in retrospective study. Bulletin of the Veterinary Institute in Pulawy 53, 589–595. https://doi.org/10.13140/RG.2.1.2889.2008

Sonntag, M., Mühldorfer, K., Speck, S., Wibbelt, G., Kurth, A., 2009. New adenovirus in bats, Germany. Emerg Infect Dis 15, 2052–2055. https://doi.org/10.3201/eid1512.090646

Speranskaya, A.S., Artiushin, I. V., Samoilov, A.E., Korneenko, E. V., Khabudaev, K. V., Ilina, E.N., Yusefovich, A.P., Safonova, M. V., Dolgova, A.S., Gladkikh, A.S., Dedkov, V.G., Daszak, P., 2023. Identification and Genetic Characterization of MERS-Related Coronavirus Isolated from Nathusius’ Pipistrelle (*Pipistrellus nathusii*) near Zvenigorod (Moscow Region, Russia). Int J Environ Res Public Health 20. https://doi.org/10.3390/ijerph20043702

Takumi, K., Lina, P.H.C., van der Poel, W.H.M., Kramps, J.A., van der Giessen, J.W.B., 2009. Public health risk analysis of *European bat lyssavirus* infection in The Netherlands. Epidemiol Infect 137, 803–809. https://doi.org/10.1017/S0950268807000167

Tóth, G.E., Hume, A.J., Suder, E.L., Zeghbib, S., Ábrahám, Á., Lanszki, Z., Varga, Z., Tauber, Z., Földes, F., Zana, B., Scaravelli, D., Scicluna, M.T., Pereswiet-Soltan, A., Görföl, T., Terregino, C., De Benedictis, P., Garcia-Dorival, I., Alonso, C., Jakab, F., Mühlberger, E., Leopardi, S., Kemenesi, G., 2023. Isolation and genome characterization of Lloviu virus from Italian Schreibers’s bats. Sci Rep 13. https://doi.org/10.1038/s41598-023-38364-7

Troupin, C., Picard-Meyer, E., Dellicour, S., Casademont, I., Kergoat, L., Lepelletier, A., Dacheux, L., Baele, G., Monchâtre-Leroy, E., Cliquet, F., Lemey, P., Bourhy, H., 2017. Host genetic variation does not determine spatio-temporal patterns of European bat 1 lyssavirus. Genome Biol Evol 9, 3202–3213. https://doi.org/10.1093/gbe/evx236

Van Der Poel, W.H.M., Van Der Heide, R., Verstraten, E.R.A.M., Takumi, K., Lina, P.H.C., Kramps, J.A., 2005. European bat lyssaviruses, the Netherlands. Emerg Infect Dis 11, 1854–1859. https://doi.org/10.3201/eid1112.041200

Van Gucht, S., Verlinde, R., Colyn, J., Vanderpas, J., Vanhoof, R., Roels, S., Francart, A., Brochier, B., Suin, V., 2013. Favourable outcome in a patient bitten by a rabid bat infected with the *European bat lyssavirus-1*. Acta Clin Belg 68, 54–58. https://doi.org/10.2143/ACB.68.1.2062721

Vázquez-Morón, S., Avellón, A., Echevarría, J.E., 2006. RT-PCR for detection of all seven genotypes of *Lyssavirus* genus. J Virol Methods 135, 281–287. https://doi.org/10.1016/j.jviromet.2006.03.008

Vázquez-Morón, Sonia, Ibáñez, C., Juste, J., Echevarria, J.E., 2006. EBLV1 circulation in natural bat colonies of *Eptesicus serotinus*: A six year survey. Dev Biol (Basel) 125, 257–261.

Vázquez-Morón, S., Juste, J., Ibáñez, C., Aznar, C.J., Ruiz-Villamor, E., Echevarría, J.E., 2008a. Asymptomatic rhabdovirus infection in meridional serotine bats (*Eptesicus isabellinus*) from Spain. Dev Biol (Basel) 131, 311–316.

Vázquez-Morón, S., Juste, J., Ibáñez, C., Berciano, J.M., Echevarría, J.E., 2011. Phylogeny of European bat lyssavirus 1 in *Eptesicus isabellinus* bats, Spain. Emerg Infect Dis 17, 520–523. https://doi.org/10.3201/eid1703.100894

Vázquez-Morón, S., Juste, J., Ibáñez, C., Ruiz-Villamor, E., Avellón, A., Vera, M., Echevarría, J.E., 2008b. Endemic circulation of European bat lyssavirus type 1 in serotine bats, Spain. Emerg Infect Dis 14, 1263–1266. https://doi.org/10.3201/1408.080068

Vidovszky, M.Z., Boldogh, S.A., 2011. Detection of adenoviruses in the Northern Hungarian bat fauna. Magyar Állatorvosok 133, 747–753.

Vidovszky, M.Z., Kohl, C., Boldogh, S., Görföl, T., Wibbelt, G., Kurth, A., Harrach, B., 2015. Random sampling of the Central European bat fauna reveals the existence of numerous hitherto unknown adenoviruses. Acta Vet Hung 63, 508–525. https://doi.org/10.1556/004.2015.047

Wibbelt, G., Kurth, A., Yasmum, N., Bannert, M., Nagel, S., Nitsche, A., Ehlers, B., 2007. Discovery of herpesviruses in bats. Journal of General Virology 88, 2651–2655. https://doi.org/10.1099/vir.0.83045-0

Wilson, D. E., & Mittermeirer, R. A. (Eds.). (2019). Bats, Handbook of the Mammals of the World Vol. 9 (1st ed.). Lynx Edicions, Barcelona.

Wiederkehr, M.A., Qi, W., Schoenbaechler, K., Fraefel, C., Kubacki, J., 2022. Virus diversity, abundance, and evolution in three different bat colonies in Switzerland. Viruses 14. https://doi.org/10.3390/v14091911
